# Characterization of ALTO-encoding circular RNAs expressed by Merkel cell polyomavirus and trichodysplasia spinulosa polyomavirus

**DOI:** 10.1371/journal.ppat.1009582

**Published:** 2021-05-17

**Authors:** Rong Yang, Eunice E. Lee, Jiwoong Kim, Joon H. Choi, Elysha Kolitz, Yating Chen, Clair Crewe, Nicholas J. H. Salisbury, Philipp E. Scherer, Clay Cockerell, Taylor R. Smith, Leslie Rosen, Louisa Verlinden, Denise A. Galloway, Christopher B. Buck, Mariet C. Feltkamp, Christopher S. Sullivan, Richard C. Wang

**Affiliations:** 1 Department of Dermatology, UT Southwestern Medical Center, Dallas, Texas, United States of America; 2 Quantitative Biomedical Research Center, Department of Population and Data Sciences, UT Southwestern Medical Center, Dallas, Texas, United States of America; 3 Department of Molecular Biosciences, University of Texas, Austin, Texas, United States of America; 4 Touchstone Diabetes Center, Department of Internal Medicine, the UT Southwestern Medical Center, Dallas, Texas, United States of America; 5 Division of Human Biology, Fred Hutchinson Cancer Research Center, Seattle, Washington, United States of America; 6 Department of Dermatology, Louisiana State University Health Sciences Center, New Orleans, Louisiana, United States of America; 7 Department of Dermatology, Ghent University Hospital, Ghent, Belgium; 8 Lab of Cellular Oncology, NCI, NIH, Bethesda, Maryland, United States of America; 9 Department of Medical Microbiology, Leiden University Medical Center, Leiden, Netherlands; 10 Harold C. Simmons Cancer Center, UT Southwestern Medical Center, Dallas, Texas, United States of America; University of Wisconsin Madison School of Medicine and Public Health, UNITED STATES

## Abstract

Circular RNAs (circRNAs) are a conserved class of RNAs with diverse functions, including serving as messenger RNAs that are translated into peptides. Here we describe circular RNAs generated by human polyomaviruses (HPyVs), some of which encode variants of the previously described alternative large T antigen open reading frame (ALTO) protein. Circular ALTO RNAs (circALTOs) can be detected in virus positive Merkel cell carcinoma (VP-MCC) cell lines and tumor samples. CircALTOs are stable, predominantly located in the cytoplasm, and N^6^-methyladenosine (m^6^A) modified. The translation of MCPyV circALTOs into ALTO protein is negatively regulated by MCPyV-generated miRNAs in cultured cells. MCPyV ALTO expression increases transcription from some recombinant promoters in vitro and upregulates the expression of multiple genes previously implicated in MCPyV pathogenesis. MCPyV circALTOs are enriched in exosomes derived from VP-MCC lines and circALTO-transfected 293T cells, and purified exosomes can mediate ALTO expression and transcriptional activation in MCPyV-negative cells. The related trichodysplasia spinulosa polyomavirus (TSPyV) also expresses a circALTO that can be detected in infected tissues and produces ALTO protein in cultured cells. Thus, human polyomavirus circRNAs are expressed in human tumors and infected tissues and express proteins that have the potential to modulate the infectious and tumorigenic properties of these viruses.

## Author summary

Human polyomaviruses (HPyV) have been linked to diseases including Merkel cell carcinoma (MCC), a skin cancer caused by Merkel cell polyomavirus (MCPyV), and skin and hair eruptions caused by trichodysplasia spinulosa polyomavirus (TSPyV). We discover that HPyVs generate single-stranded, circular RNAs (circRNAs) in affected tissues. Both MCPyV and TSPyV generate circRNAs encompassing part of the early region (circALTOs) that can be translated to the alternative large T antigen open reading frame (ALTO) protein in cultured cells. Previously described microRNAs (miRNAs) generated by MCPyV have the ability to inhibit the expression of ALTO. MCPyV circALTOs are enriched in extracellular vesicles produced by MCC cell lines and cells that express circALTO. Finally, we discover that MCPyV ALTO protein enhances transcription from some recombinant promoters and promotes the expression of multiple host cell genes previously implicated in MCPyV pathogenesis. Thus, HPyVs generate circRNAs, including circALTOs that are translated to ALTO protein, and MCPyV ALTO has the ability to regulate transcription.

## Introduction

About a dozen polyomaviruses infect humans [1]. Merkel cell carcinoma (MCC) is a rare, deadly skin cancer linked to Merkel cell polyomavirus (MCV or MCPyV) infection in 80% of cases [2]. In addition, trichodysplasia spinulosa polyomavirus (TSPyV) causes a primary infection that presents as eruptions of skin spicules and follicular papules in immunosuppressed patients [3–6]. The small, double-stranded DNA genome of polyomaviruses includes early, late, and non-coding control regions [7]. The polyomavirus early region (ER) encodes for small T-antigen (sT) and large T-antigen (LT), while the late region contains late structural proteins VP1 and VP2 [8–10]. Moreover, MCPyV has been reported to encode a microRNA (miRNA) that lies antisense to the early transcripts [11,12]. In a subset of *Alphapolyomaviruses*, including MCPyV and TSPyV, the early region has been shown to generate several variably spliced transcripts, including a transcript that encodes a protein in an alternate reading frame from large T antigen (ALTO) [13,14]. ALTO shares a common evolutionary origin with middle T antigen (MT) of murine polyomavirus (MPyV) [13,15]. Although MPyV MT has transforming activity and has been a useful tool to study cellular transformation [16], the precise function of human ALTO proteins remain unclear.

Circular RNAs (circRNAs) are covalently closed single-stranded RNAs that are most commonly produced by non-canonical 3’-5’ ligation or splicing of linear RNA [17,18]. More than 40 years ago, viroids were the first recognized single-stranded circular RNA species [19]. Later, RNAs with circular structure were observed in eukaryotic cell lines by electron microscopy (EM) [20], and were then found in all major domains of life [21,22]. Recent improvements in high-throughput RNA sequencing and computational approaches have revealed circRNAs to be regulated and abundant [23,24]. In addition to the endogenous circRNAs generated by eukaryotic cells, an increasing number of viruses have been found to encode for circRNAs. Epstein-Barr virus (EBV), Kaposi’s sarcoma herpesvirus (KSHV), and human papillomaviruses (HPVs) have all been demonstrated to generate circRNAs [25–29]. The HPV-derived circRNA, circE7, encodes for the E7 oncoprotein and is essential for the transformed growth of CaSki cervical carcinoma cells [30].

CircRNAs exert their functions in eukaryotic cells through a variety of mechanisms. They were first recognized to act as miRNA sponges [31,32], such as ciRS-7/CDR1as, which acts as a miRNA sponge or decoy for miR-7 [32]. Some circRNAs bind to RNA-binding proteins (e.g. HuR, RNA Pol II) to influence transcription and translation [33,34]. Numerous circRNAs can be translated to functional proteins [30,35]. CircRNAs have been found to be resistant to degradation and also enriched in extracellular vesicles, including exosomes and virions, suggesting ways in which circRNAs might exhibit distinct functions compared to their linear counterparts [28,36,37].

Here we describe the identification of circular RNAs encoded by human polyomaviruses. MCPyV has two circRNAs in the early region encompassing the ALTO gene (circALTO1 and circALTO2) that could be detected in VP-MCC cell lines and patient tumors. TSPyV encodes a smaller circALTO, which could be efficiently generated in vitro and detected in infected tissues from TS patients. The characterization of circALTO revealed a possible function for MCPyV ALTO, suggesting novel ways in which circRNAs could influence the infectious and transforming activities of HPyVs.

## Results

### Identification of Merkel cell polyomavirus circRNAs

**Fig 1:**
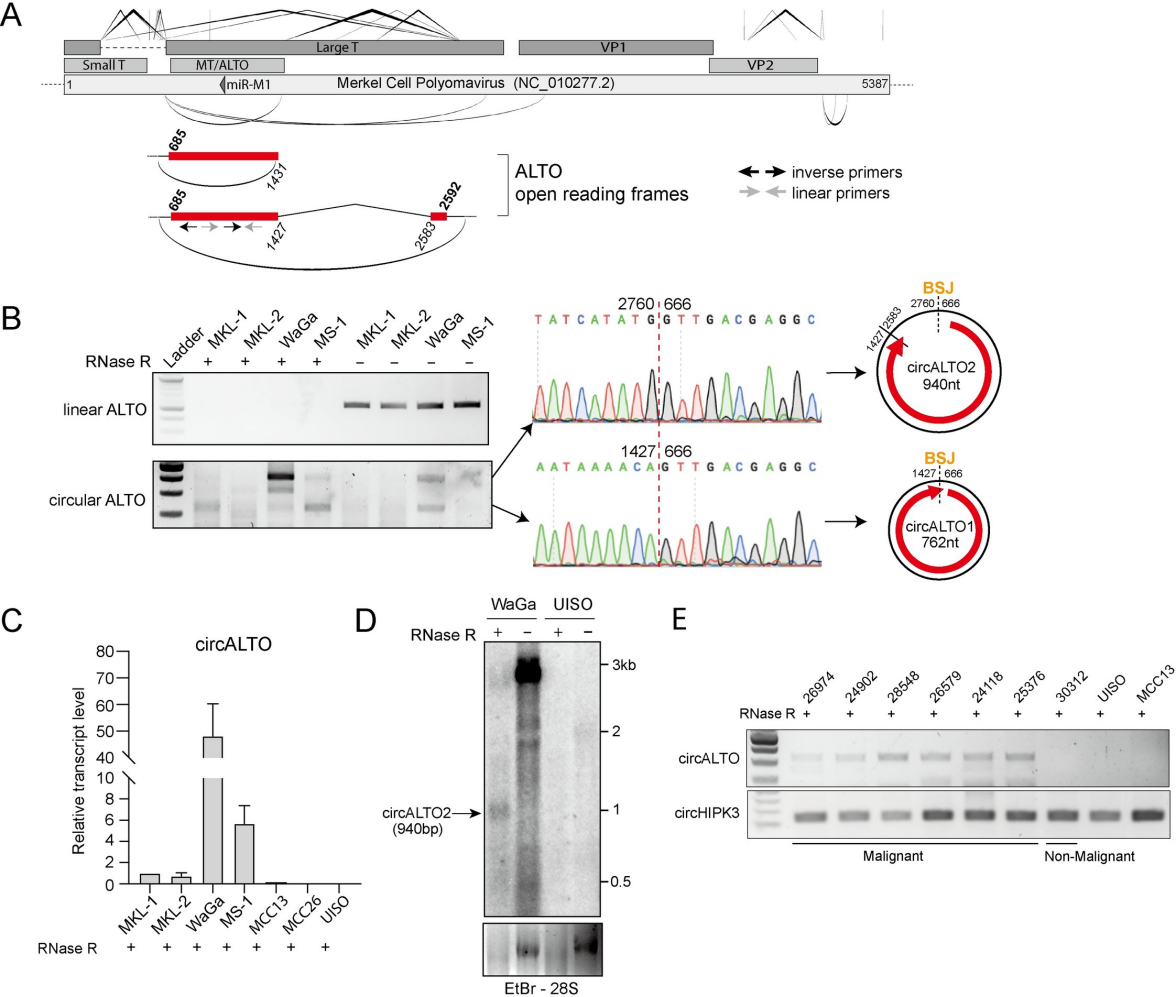
Identification of Merkel cell polyomavirus circRNAs. (A) (Top panel) Schematic representation of potential circRNAs identified by the vircircRNA for MCPyV. V-shaped lines above the map indicate forward splicing events. Elliptical arcs below the map indicate predicted backsplicing. (Bottom panel) Red rectangles indicate ALTO open reading frames. Bold numbers above the map indicate the first base of ALTO start and stop codons, italic numbers below the map indicate the positions of exon boundaries. (B) (Left panel) RT-PCR analysis of four VP-MCC cell lines (MKL1, MKL-2, MS-1 and WaGa) with and without RNase R treatment. (Middle panel) Sanger sequencing of inverse PCR products from WaGa cells confirmed the backsplice junction of the predicted circRNAs. (Right panel) Schematic of the two different circALTO forms, we termed circALTO1 and circALTO2 based on the product length. (C) qRT-PCR analysis for circALTOs from VP-MCC (MKL-1, MKL-2, MS1, and WaGa) and VN-MCC (MCC13, MCC26 and UISO) after RNase R treatment. *ACTB* served as the normalization control. Values are the mean of three technical replicates; bars represent standard deviation. (D) Northern blot of total RNA after with and without RNase R treatment from VP-MCC cell line WaGa and VN-MCC cell line UISO using an ALTO-specific probe. Arrow indicates RNase R resistant band consistent with circALTO2. (E) Endpoint PCR confirmed the presence of circALTO2 in patient MCC tumors (upper panel), but not a non-malignant skin control or VN-MCC cell lines. *circHIPK3* served as a control for RNA integrity (bottom panel).

To determine whether MCPyV might generate circRNA, we used the previously described vircircRNA pipeline on RNA-Seq datasets of MCPyV infected cells (https://github.com/jiwoongbio/vircircRNA) [30]. Three potential circRNAs in the early region were predicted to share the same 5’ splice site, but distinct 3’ splice site, and all three encompassed both the previously described MCPyV-encoded miRNA docking sites (complementary to the miRNA locus) and the ALTO/MT ORF (Fig 1A, Table A in S1 Fig). Additional potential circRNAs were also identified upstream of the VP2 minor capsid protein ORF in a region where some polyomaviruses encode an agnoprotein gene.

To confirm the presence of circRNA transcripts, we used inverse PCR on four VP-MCC cell lines, MKL-1, MKL-2, MS-1, and WaGa. Inverse PCR products could be identified for the predicted circRNAs in the early region, but not adjacent to the late region, likely because the expression of late genes is typically down-regulated in MCC tumors (Fig 1B). We chose to focus on the characterization of the potential circRNAs in the early region (circALTO).

RNase R, an exonuclease that degrades most linear RNAs, was used to distinguish between linear RNAs arising from multiple passes around the circular viral genome and RNAs circularized by backsplicing. Total RNA from four VP-MCC cell lines was subjected to inverse RT-PCR targeting circALTO. PCR products remained detectable after RNase R digestion, indicating the presence of circular RNAs (Fig 1B). The RNase R resistant inverse PCR products from WaGa cells were then sequenced. The slower-migrating band, circALTO2, represents a 940 nucleotide (nt) circRNA with a ‘backspliced’ ALTO ORF that encompasses an additional canonical splice that has previously been described for linear ALTO mRNAs. The faster-migrating, circALTO1, is a 762nt circRNA containing most of the ALTO ORF (Fig 1B). Other intermediate-sized inverse RT-PCR bands observed in VP-MCC cells were found to contain insertions not derived from the MCPyV genome (S1B Fig). Next, we compared levels of circular ALTO RNAs from RNA prepared from VP-MCC and virus-negative (VN)-MCC lines (MCC13, MCC26, and UISO) with RNase R treatment. We designed the PCR primers in a region shared by both circALTO1 and circALTO2. Both endpoint and qPCR revealed that circALTO could be detected in VP-MCC, but not VN-MCC cells (Figs 1C and S1C). The presence of circALTO2 in RNase R-treated total RNA preparations of WaGa cells was confirmed by northern blotting (Fig 1D). Finally, we next tested for the expression of circALTO in patient tumors (Fig 1E and S1E, and Table D in S1 Fig). Total RNA from frozen tumors was subjected to RNAse R treatment and then endpoint RT-PCR. A previously described abundant cellular circRNA, circHIPK3, was present in all samples [31]; circALTO2 was detected in all 6 VP-MCC tumors, but not in a non-malignant tissue control or VN-MCC lines (Fig 1E). Despite its detection in VP-MCC cell lines, circALTO1 could not be detected in the tumor tissues suggesting that the abundance of the circALTO isoforms might differ in vivo.

### Characterization of circALTO

**Fig 2:**
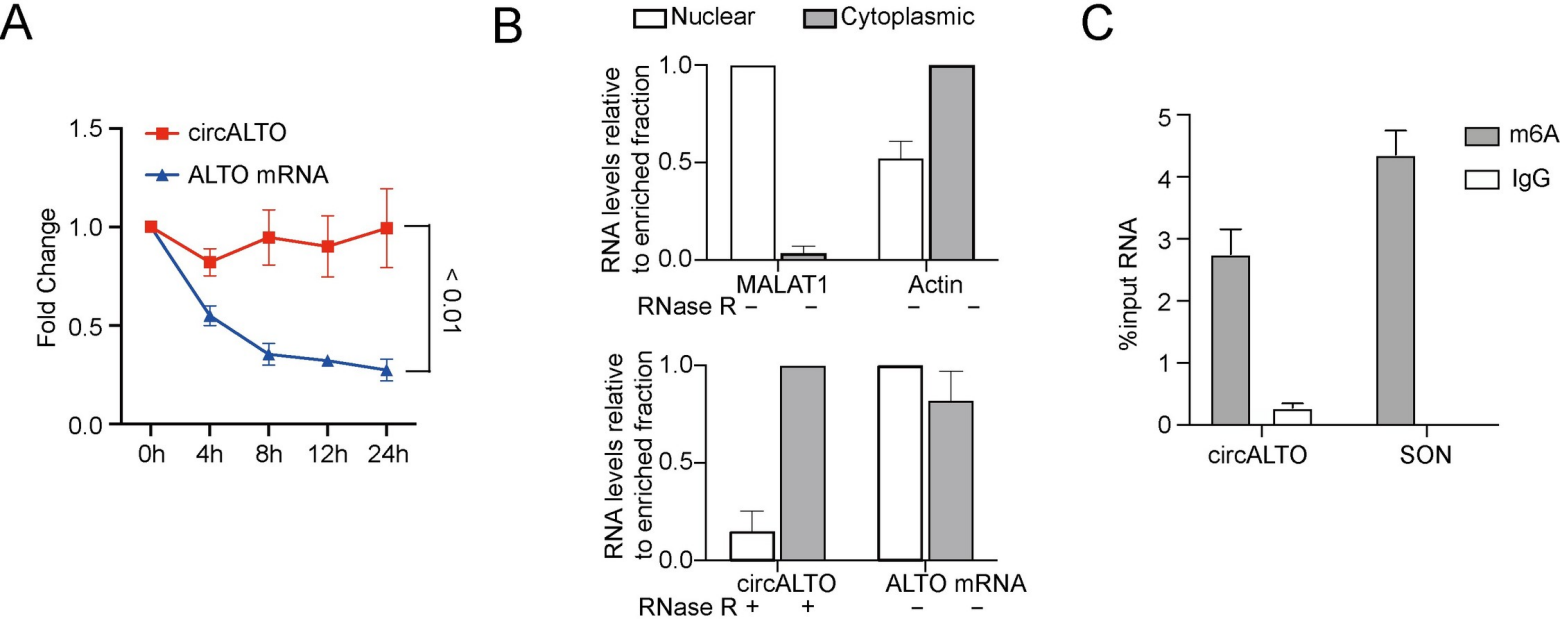
Characterization of circALTO. (A) The expression of circALTOs and its linear mRNA counterparts after treatment with Actinomycin D at the indicated time points in WaGa cells by qRT–PCR analysis. Both circALTO and linear ALTO were normalized to *18S* and then compared to levels at the pre-treatment (0 h) time point. Error bars calculated the SD from 3 biological replicates. The *P* value was determined using two-tailed t-test. (B) WaGa were fractionated and analyzed by qRT-PCR. *MALAT1* and *ACTB* served as nuclear and cytoplasmic fractionation controls respectively. Values are normalized to the enriched fraction. Error bars were calculated from 3 biological replicates. (C) qRT-PCR of m^6^A or IgG control RNA immunoprecipitation (RIP) of VP-MCC cell lines WaGa. SON, m^6^A RNA IP control. Error bars represent SD (n = 3 biological replicates).

Circular RNAs have been reported to be more stable than linear RNA. Indeed, after treatment with the transcriptional inhibitor Actinomycin D [31], a time course qRT-PCR analysis showed that circALTOs were significantly more stable than linear ALTO mRNAs, with a half-life exceeding 24 hours in WaGa cells (Fig 2A). Next, we investigated the localization of circALTO in WaGa cells. Cellular fractionation analyses showed that circALTOs mainly localized to cytoplasm while the majority of linear ALTO RNA localized to the nucleus (Fig 2B). Thus, circALTOs are very stable and largely localized to the cytoplasm. Because N^6^-methyladenosine (m^6^A) modifications have been reported to be abundant on circRNAs and have been reported to promote m^6^A reader protein binding, splicing, and cap-independent translation [38–40], we performed m^6^A RNA immunoprecipitation to determine whether the circALTO RNAs might be modified. In WaGa cells, we found both SON, a positive control mRNA, and circALTO were pulled down by m^6^A antibody precipitation, but not an IgG control antibody (Fig 2C).

### Confirmation using recombinant circALTO expression vectors

To evaluate potential functions of circALTO, we generated expression vectors encompassing the entire backspliced region of ALTO gene (S2A Fig). All constructs include Quaking (QKI) protein-binding sites which have been previously been shown to enhance the circularization of RNAs [41] and some constructs also contain an amino(N)-terminal 3xFlag epitope tag (S2A Fig). The circALTO2 construct contains an additional exon which includes an in-frame stop codon. In contrast, circALTO1 does not naturally contain an in-frame stop codon when circularized, and thus appears to encode for a circular ORF capable of ‘infinite’ rolling circle translation (S2A Fig). The circALTO constructs were transfected into HEK293T cells, and RNase R-resistant circRNAs were detected by RT-PCR from both WT and epitope-tagged circALTO (S2B Fig, left). Sanger sequencing of the RT products confirmed that the circALTO construct generated the expected backsplice junctions (S2B Fig, right). As with the circular MCPyV genome and integrated tandem repeats of the viral genome, read-through transcription of the entire pCDNA3.1-based plasmid followed by forward splicing between distinct copies of the ALTO region could theoretically generate “false backsplice” junctions. Indeed, primers amplifying the CMV promoter region could be detected by RT-PCR, indicating that readthrough transcription of the pCDNA vector could occur. However, readthrough transcription products could no longer be detected after RNase R treatment while the relative detection of circALTO backsplice junctions increased after RNase R treatment (Figs 1B and S2C). Finally, northern blots confirmed that an RNase R resistant RNA of the expected size of circALTO2 was generated by the circALTO2 construct (S2D Fig). CircALTO1 could not be reliably distinguished above background signals, possibly due to its smaller size. Fractionation and quantitation of nuclear and cytoplasmic circALTO from 293T cells transfected with circALTO1 (S2E Fig) demonstrated that circALTO1 was enriched in the cytoplasmic fraction, consistent with the findings from WaGa cells (Fig 2B).

### CircALTOs do not sponge MCPyV miR-M1 in cultured cells

**Fig 3:**
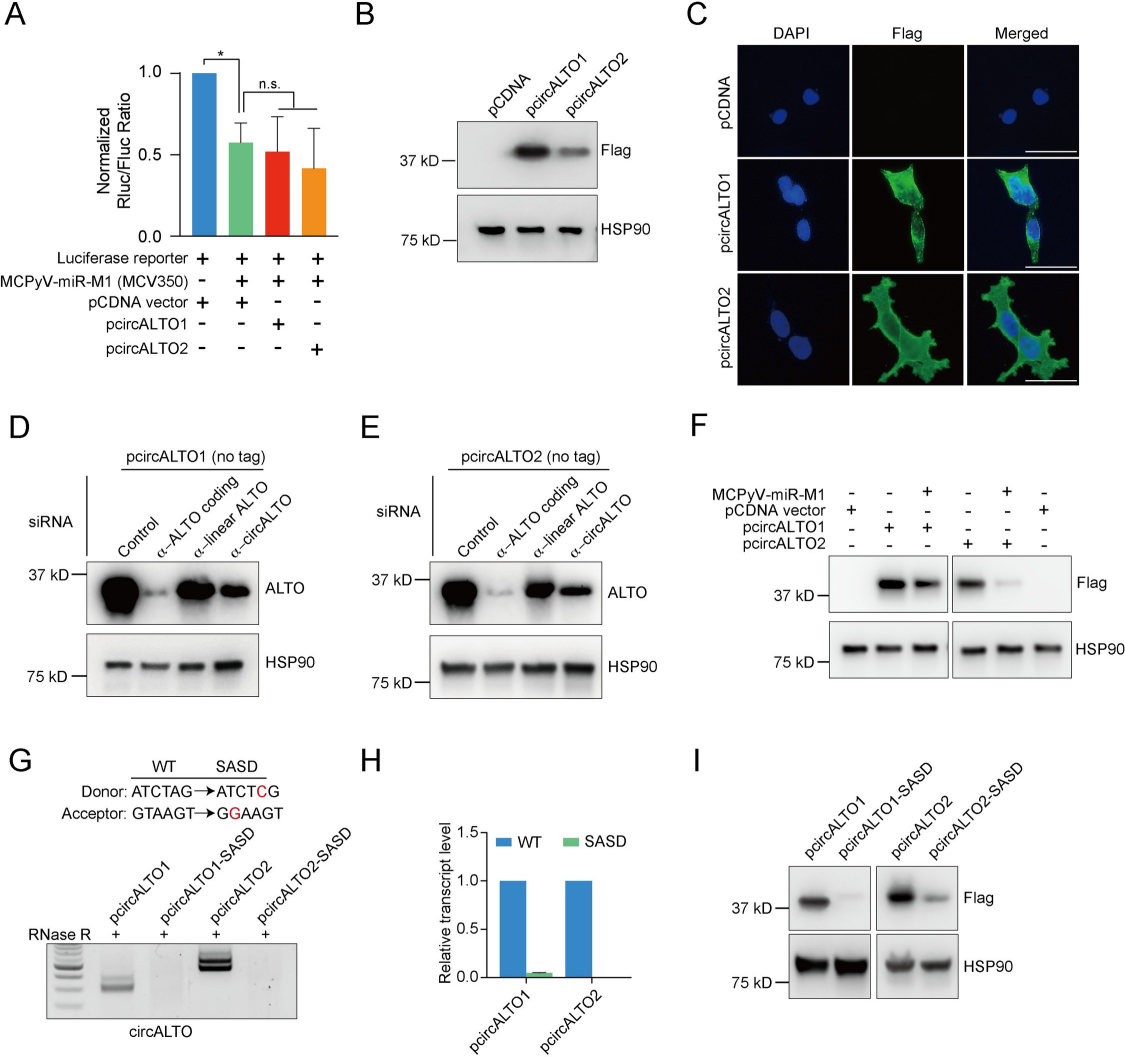
MCPyV circALTO can be translated. (A) CircALTOs do not inhibit MCPyV miRNA function in vitro. 293 cells were transfected with *Renilla* luciferase reporter with a 300 nucleotide region complementary to the MCPyV miRNA and the indicated plasmids including a MCPyV-miRNA-M1 expression plasmid (derived from isolate MCV350), pCDNA3.1 control vector, pCDNA circALTO1 (pcircALTO1) or pCDNA circALTO2 (pcircALTO2) expression plasmid. Firefly luciferase served as a transfection control and *Renilla* luciferase levels are plotted normalized relative to firefly luciferase levels (n = 2 biological replicates). The *P* value was determined by unpaired two-tailed t-test. (B) Western blot (WB) for FLAG from 293T cells co-transfected with a pCDNA vector control plasmid or pCDNA3.1-Flag-circALTO1/2. HSP90, loading control. (C) 293T cells were transfected with vector, the Flag-circALTO1/2 plasmids. After 48 hours, cells were fixed and stained for FLAG (green) and DAPI (blue). Scale bar = 50 μm. (D) WB for ALTO protein from 293T cells co-transfected with circALTO1 (untagged) and siRNAs targeting the body of the ALTO ORF, a segment unique to linear ALTO transcripts, or an siRNA targeting the unique junction of circALTO1. (E) WB for ALTO protein from 293T cells co-transfected with circALTO2 (untagged). (F) Immunoblotting analysis of 293T cells were transfected with empty vector, pCDNA-circALTO1 (Flag), or pcircALTO2 (Flag) with or without MCPyV miRNA expression vector. Co-transfection with the MCPyV miRNA expression vector results in decreased expression of ALTO. (G) Endpoint inverse RT-PCR detection of analysis for circALTO from 293T cells transfected with pcircALTO1/2 (WT) and the indicated constructs harboring splice sites mutations (SASD) (see S2A Fig). (H) Inverse qRT-PCR analysis of circALTO from cells with WT constructs and constructs harboring SASD mutant (see S2A Fig). *ACTB* served as the internal control. Error bar represents the standard deviation of 3 independent experiments. (I) Western blots for Flag-ALTO from cells with WT constructs and constructs harboring the SASD mutation. HSP90, loading control. The *P* value was determined by unpaired, two-tailed t-test, *<0.05, **<0.01.

Many circRNAs function as microRNA (miRNA) “sponges” [32,42,43], and the MCPyV ALTO locus encodes for miRNAs, miR-M1-3p and miR-M1-5p, complementary to the circALTO transcript [11,12]. Therefore, we tested whether circALTOs might bind and inhibit the MCPyV miRNA. We used the circALTO constructs together with a luciferase reporter construct to investigate the ability of circALTOs to bind to MCPyV miRNA in transfected cells. A reporter plasmid that contains Renilla luciferase fused to a 300 nucleotide region of the MCPyV early transcript shows decreased luciferase expression when co-transfected with an MCPyV miR-M1 (MCC350 miRNA variant) expressing plasmid [11]. Using these plasmids, we tested whether circALTO might be able to sponge miR-M1-5p/3p and thereby rescue miR-M1 mediated silencing of the early region. When we co-transfected a MCPyV miR-M1 expression vector and a circALTO1/2 expression vector, luciferase expression was not rescued compared to the HEK293 cells transfected with MCPyV miRNA alone (Fig 3A). This inability to rescue luciferase expression was confirmed in HEK293T cells, and was also observed when we assayed a sequence variant MCPyV miRNA (MCC339) (S3A and S3B Fig). These results suggest that the circALTO does not function as an inhibitor for MCPyV miR-M1.

### CircALTOs can be translated

We next investigated whether circALTOs might be translated. Transfection of both native untagged or Flag-tagged circALTO expression vectors in 293T cells resulted in the production of proteins whose size was consistent with the previously described MCPyV ALTO protein (Fig 3B, 3D and 3E). Immunofluorescence assays showed that cells transfected with circALTO1/2 generated Flag-ALTO proteins which were localized to cytoplasmic foci (Fig 3C). To confirm that ALTO was encoded by the circular, rather than linear, ALTO RNAs, we used small interfering RNAs (siRNAs) to target the expression of ALTO RNA isoforms in circALTO1/2 transfected cells. Several siRNAs were designed for both circALTO1 and circALTO2 constructs: one targeting the circALTO1 or 2 backsplice junction (BSJ), a second targeting a linear region outside the backsplice exon, and a final siRNA targeting sequences shared by both the circular and linear RNAs within the ALTO ORF coding region. The efficiency of siRNA knockdown was confirmed by qRT-PCR analysis; siRNAs against the BSJ sequences specifically knocked down circALTO1/2, the siRNA against the linear sequences only significantly knocked down the linear ALTO transcript, and the siRNA targeting the ALTO coding sequences significantly decreased both circular and linear ALTO transcript levels (S4A and S4C Fig). Notably, the BSJ- and ALTO-targeting siRNAs strongly downregulated ALTO expression, whereas specific knockdown of the linear transcript only modestly impacted ALTO expression levels (Figs 3D, 3E, S4B and S4D). Similar results were obtained for Flag-epitope tagged ALTO constructs. In addition, in immunofluorescence experiments, cells co-transfected circALTO1/2 plasmids and BSJ-specific siRNAs showed marked reductions in Flag staining compared to circALTO1/2 control transfections (S4E Fig). The results indicate that a majority of ALTO protein expression in this system is attributable to circALTO RNA.

The annealing of fully complementary miRNA’s can result in the Ago2-dependent cleavage and degradation of mRNA [44]. While circALTO1/2 did not appear to possess an ability to sponge miR-M1, we tested whether circALTO1/2 stability might be impacted by miR-M1-5p/3p binding. We co-transfected the circALTO1/2 and miR-M1 expression plasmids and found that ALTO expression levels were markedly decreased compared to cells transfected with circALTO1/2 alone (Fig 3F). Thus, in these in vitro assays, rather than sponging miR-M1, circALTO appears to be negatively regulated by miR-M1 in this system.

N^6^-methyladenosine (m^6^A) modifications have been implicated as a common feature of circRNA expression [38]. To assess the role of m^6^A modification in circALTO expression, we used siRNA to knock down Mettl3/14, the conserved m^6^A methyltransferase complex. Transfection of RNAs targeting the Mettl3/14 complex reduced Mettl3 levels and also significantly reduced circALTO transcript level (S4F and S4G Fig). Thus, m^6^A modifications are likely required for efficient backsplicing or stability of circALTO isoforms. Finally, to confirm that backsplicing was necessary for the expression and translation of circALTO1/2, we generated circALTO constructs with mutations in the splice acceptor and splice donor sites (SASD) (Fig 3G). As expected, both endpoint and qRT-PCR (Fig 3G and 3H) analyses did not show detectable levels of circALTO transcripts from the constructs with splice site mutations. Consistent with a critical role for splicing in ALTO translation in vitro, ALTO protein levels were also markedly downregulated in the splice site mutant constructs compared with wild type control (Fig 3I). In summary, we find that circALTO1 and circALTO2 generate ALTO proteins in cultured cells.

### CircALTOs increase expression from the human cytomegalovirus (CMV) immediate early promoter and SV40 early/late promoters

**Fig 4:**
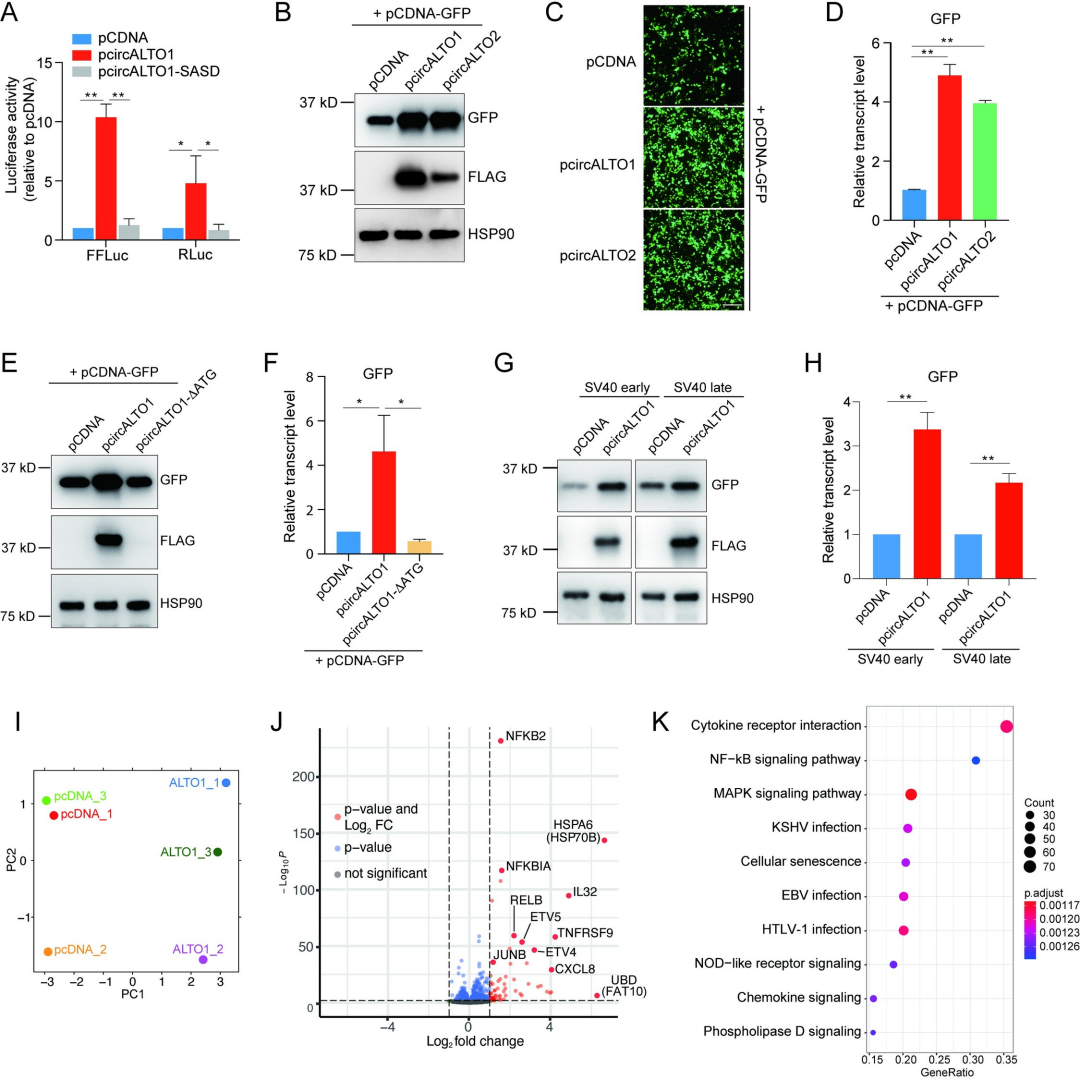
MCPyV circALTO increases transcription from specific promoters and can modulate host cell gene expression. (A) 293 cells were co-transfected with *Renilla* luciferase reporter or Firefly luciferase reporter and with either pCDNA vector control, pcircALTO1, or pcircALTO1-SASD (n = 3 biological replicates). The *P* value was determined by unpaired two-tailed t-test. (B) Western blot for GFP and FLAG from 293T cells which were co-transfected with pCDNA-GFP and indicated plasmids including pCDNA control vector, pcircALTO1 (Flag), or pircALTO2 (Flag) plasmids. HSP90 is the loading control. (C) 293T cells were co-transfected with pCDNA-GFP vector and indicated plasmids. IF images are representative of GFP expression after 48 hours. Scale bar = 200 μm. (D) qRT-PCR analysis for GFP from cells with pCDNA-GFP constructs and indicated plasmids. Expression was normalized against *ACTB*. Error bar representative of 3 independent experiments. (E) A construct in which all potential ATG start codons have been mutated, circALTO1-ΔATG, was generated. Western blot for GFP and FLAG from 293T cells were transfected with pCDNA-GFP reporter vector and pCDNA control vector, Flag-circALTO1, and pcircALTO1-ΔATG plasmids. HSP90 is the loading control. (F) qRT-PCR analysis for GFP transcripts from 293T cells as described in (E). Expression was normalized against *ACTB*. Error bars = SD from one biological replicate. Results are representative of 3 independent experiments. (G) Western blot for GFP and FLAG from 293T cells which were co-transfected with the SV40 early or late promoter cloned into pU-5864 and the indicated plasmids including pCDNA control vector or pcircALTO1 (Flag). HSP90 is the loading control. (H) qRT-PCR analysis for GFP from cells co-transfected with the SV40 early or late promoter cloned into pU-5864 and the indicated plasmids including pCDNA control vector or pcircALTO1 (Flag). HSP90 is the loading control. Expression was normalized against *ACTB*. Error bar representative of 3 biological replicates. (I) Principal component analysis (PCA) of pCDNA control and pcircALTO1 transcriptomes (48 hours) reveals distinct clustering of ALTO compared with vector control. (J) Volcano plot (single gene) of differentially expressed genes in pcircALTO1 compared with pCDNA vector. Significantly upregulated genes are indicated in red (log2 FC>1.5, adjusted p<0.001). A subset of genes previously implicated in viral infections are labeled. (K) KEGG pathway signatures induced in ALTO1 expressing cells compared to vector control. The *P* value was determined by unpaired, two-tailed t-test, *<0.05, **<0.01.

While MCPyV LT and sT antigens have been shown to play critical roles in viral replication and tumorigenesis, the biological functions of MCPyV ALTO proteins remain unknown. While testing circALTO in miRNA-interference assays, we noticed that co-transfection of either pcircALTO1 or pcircALTO2 increased luciferase expression from reporter plasmids encoding Renilla luciferase (RLuc) or firefly luciferase (FFLuc) under the control of the CMV immediate early promoter (Fig 4A). Increased expression of co-transfected luciferase plasmids required splicing of the ALTO1 RNA, as luciferase expression was not significantly increased when co-transfected with a pcircALTO1 plasmid containing mutations in the backsplice donor and acceptor sites (circALTO-SASD) (Fig 4A). To verify this observation, we tested a CMV promoter-driven green fluorescent protein plasmid, pCDNA-GFP, and confirmed that that the co-transfection of either pcircALTO1 or pcircALTO2 resulted in markedly increased levels of GFP expression by both Western blot (WB) and immunofluorescence (Fig 4B and 4C). Co-transfection of pcircALTO plasmids resulted in a ~4-fold increase in GFP transcript levels (Fig 4D). As the circALTO-SASD inhibits both circularization and ALTO expression, we next tested whether ALTO expression was required for the upregulation of co-transfected reporter plasmids. A mutant circALTO construct in which all potential ALTO ATG start codons had been mutated, circALTO1-ΔATG, could still efficiently circularize (S5A Fig), its ability to encode for ALTO protein was abrogated (Fig 4E). Notably, the circALTO1-ΔATG mutant was no longer able to increase the expression of GFP when co-transfected with the pCDNA-GFP reporter plasmid as assessed by both WB and qRT-PCR (Fig 4E and 4F).

To determine how ALTO might increase GFP transcript levels, we first determined whether reporter plasmid replication might be increased in 293T cells co-transfected with circALTO. However, copy number of the GFP plasmid as assessed by qPCR was not significantly increased after co-transfection with circALTO1/2 expression vectors compared to the vector control (S5B Fig). Next, we determined whether ALTO might affect reporter transcript stability. After co-transfecting cells with the GFP reporter plasmid and pCDNA control or pcircALTO expression vectors, cellular transcription was halted with Actinomycin D, and the stability of the GFP transcript was assessed by qRT-PCR. Co-transfection of circALTO did not significantly alter the abundance of GFP RNAs 12 hours after transcription was halted, and GFP RNA stability in circALTO transfected cells was instead somewhat lower after 24 hours of Actinomycin D treatment when compared to a vector transfected control (S5C Fig). Thus, ALTO does not increase GFP reporter RNA stability markedly, suggesting that ALTO might increase GFP transcription. To test whether ALTO might promote transcription in other promoters, we cloned and tested several additional human and viral promoter regions including: EF1-α, PGK, MCPyV early/late, TSPyV early/late, and SV40 early/late promoters. These promoters were assessed by WB and qRT-PCR (Figs 4G, 4H and S5D, and Table E in S5 Fig). MCPyV ALTO expression significantly enhanced expression from the SV40 early and late promoters (Table E in S5 Fig) but did not significantly enhance expression from the MCPyV promoter region. MCPyV ALTO expression also did not increase the expression of GFP transcripts from either of two recombinant human housekeeping promoters—elongation factor 1-alpha (EF1-α) or phosphoglycerate kinase 1 (PGK)—or from the early and late promoters of TSPyV.

The early region of MCPyV encoding ALTO also has the theoretical potential to encode additional reading frames, including a segment of the MCPyV LT protein that contains the pRB-interacting domain, as well as the MCPyV miRNAs. To exclude a role for cryptic translation of these additional reading frames in the transcriptional upregulation induced by pcircALTO, MCPyV ALTO expression construct, pALTOw, in which the ALTO ORF was codon-modified using an “as different as possible” silent mutagenesis strategy [45]. The pALTOw construct generated robust levels of ALTO protein and, like pcircALTO, induced marked up-regulation of GFP expression from the CMV promoter (S5F Fig). These findings further strengthen the contention that circALTO functions as a translation template and suggest that ALTO functions as a transcriptional regulator for some types of promoters.

### CircALTO enhances host cell signaling pathways previously implicated in polyomavirus pathogenesis

Because ALTO did not have a clear impact on the MCPyV early and late promoters, we next tested whether ALTO might impact host cell transcription. We performed RNA-sequencing on pCDNA vector or pcircALTO1 transfected 293T cells. Principal component analysis (PCA) of the sequencing revealed that circALTO1 had a strong and consistent effect on the transcriptional profile (Figs 4I and S5G). Consistent with the observation that ALTO could function as transcriptional upregulator for some promoters, a large number of genes were significantly upregulated by ALTO expression while very few genes were markedly downregulated (Figs 4J and S5G). Notably, a number of pathways previously implicated in MCPyV infection were amongst the most highly upregulated genes. NFKB2, RELB, and NFKBIA were all strongly induced, which suggested an activation of non-canonical, and inhibition of canonical, NF-κB signaling, which has been reported to be a critical function of the MCPyV sT antigen [46]. HSP70-6/HSP70B, an ortholog of HSP70, which has been shown to bind to MCPyV LT and promote MCPyV replication, was also significantly upregulated [47]. JunB, ETV4, and ETV5—transcription factors previously implicated in murine polyomavirus (MPyV) transcriptional regulation—were also significantly upregulated [48,49]. Finally, a number of inflammatory and anti-viral cytokines, including IL11, IL32, CXCL8, CD137 (TNFRSF9) were induced (Figs 4J and S5G). Gene Set Enrichment (GSE) analyses identified cytokine-cytokine receptor interaction, NF-kB signaling, and MAPK signaling signatures to have similarity to ALTO expression (Fig 4K). Moreover, gene sets consistent with infection by several DNA tumor viruses including KSHV, EBV, and HTLV-1 were also enriched, further establishing the relevance of ALTO expression in the pathogenesis of MCPyV (Fig 4K). We conclude that MCPyV ALTO can function as a transcriptional enhancer and suggest that it has the potential to modulate genes and pathways relevant to DNA tumor virus infections.

### Characterization of exosomes from circALTO expressing cells

**Fig 5:**
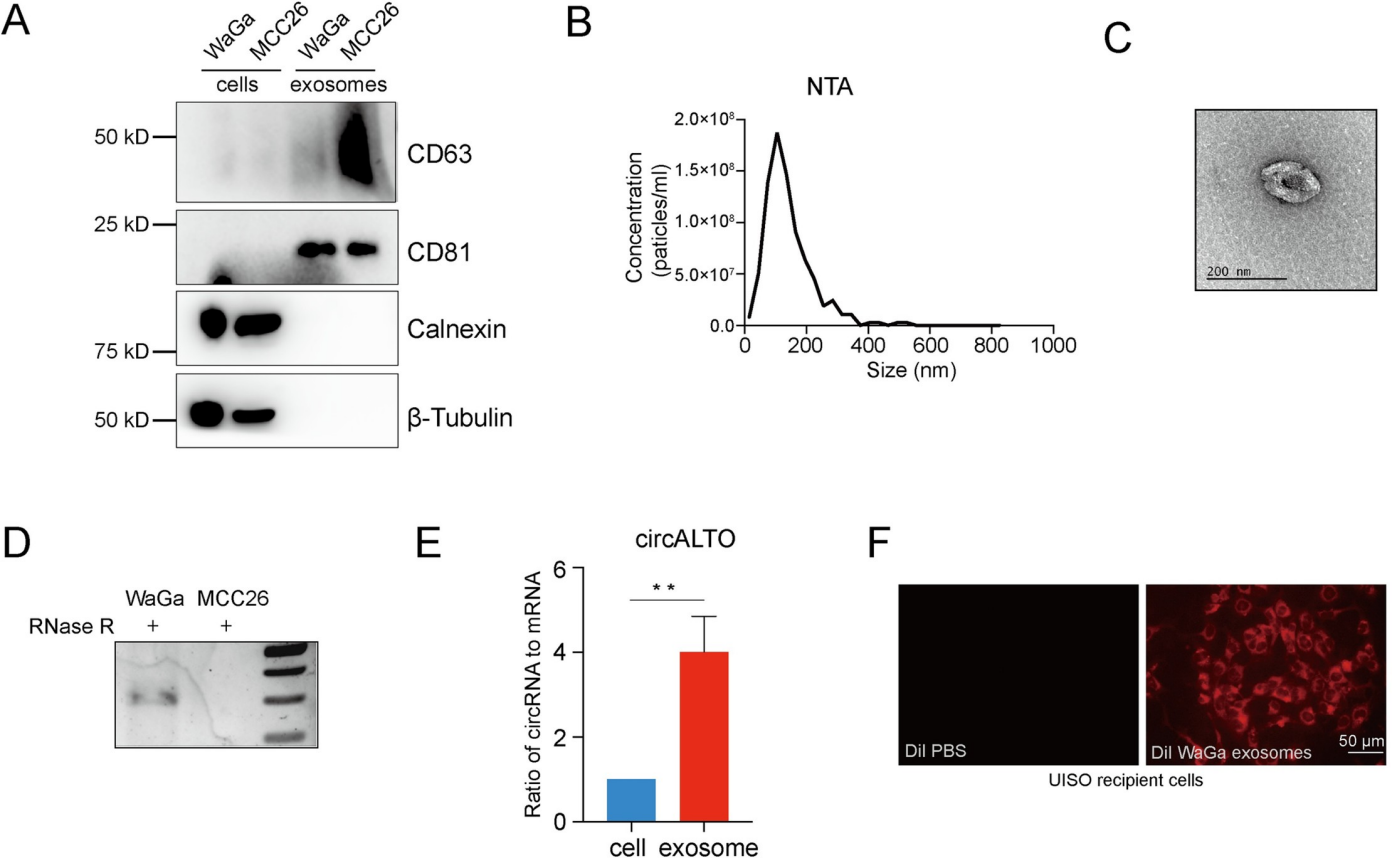
Characterization of exosomes from WaGa cells. (A) Western blot analysis of exosomal marker proteins in extracellular vesicles isolated from VP-MCC cell line WaGa and VN-MCC cell lines MCC26. CD63 and CD81 are exosomal marker proteins. The absence of calnexin and β-tubulin exclude contamination from the cytoplasm and cellular organelles. (B) Nanoparticle tracking analysis of extracellular vesicles isolated from WaGa cells are consistent with exosomes. (C) Representative TEM images of exosomes isolated from WaGa cells show the expected size and cup-shaped morphology described for exosomes. (D) RT-PCR analysis of RNase R treated total RNA prepared from exosomes of WaGa and MCC26 cells reveals the presence of circALTO in the exosomes. (E) qRT-PCR analysis the ratio of circRNA to mRNA between WaGa cells and cell-derived exosomes. Both circALTO and linear ALTO were first normalized to *ACTB*, then the ratio of circALTO:linear ALTO was set as 1. Error bars represent the SD (n = 3 biological replicates). Two-tailed t-test was performed, **<0.01. (F) Fluorescence of UISO cells incubated with DiI labeled exosomes from WaGa or Dil labeled PBS control by fluorescent microscopy. Scale bar = 50 μm.

Exosomes are 40–150 nm, membrane bound extracellular vesicles (EVs) that are enriched for specific proteins, lipids, and nucleic acids, including RNA. CircRNAs have been shown to be enriched in extracellular vesicles, including exosomes [36], and extracellular vesicles may play a role in the life cycle of polyomaviruses [50,51]. Therefore, we investigated whether circALTOs could be detected in exosomes. EVs consistent with exosomes were isolated from a VP-MCC cell line, WaGa, and a VN-MCC cell line, MCC26 through differential ultracentrifugation. To confirm the purity of the EV isolation, purified EVs were subjected to immunoblot analysis to identify the presence of common exosome associated protein markers, including CD63 and CD81. EVs isolated from WaGa and MCC26 both expressed classical exosomal markers including CD63 and CD81, but did not show evidence of contamination by cytoplasmic proteins (β-tubulin) or intracellular membranes (calnexin), which were readily detected in whole cell lysates (Fig 5A). Purified EVs were also analyzed by nanoparticle tracking analysis (NTA). NTA of EVs isolated from WaGa and MCC26 cells showed a size distribution expected for exosomes (Figs 5B and S6A) [52]. Finally, negative stain transmission electron microscopy (TEM) of WaGa EVs showed round and cup-shaped vesicles ranging from 50-150nm in size (Fig 5C).

Endpoint RT-PCR from RNA prepared from WaGa and MCC26 EVs revealed that circALTO2 could be detected in WaGa, but not MCC26 EVs (Fig 5D). Because circRNAs have been shown to be enriched in exosomes relative to their levels in the cell, we performed qRT-PCR analysis to measure the ratio of circular to linear ALTO RNA from WaGa cells. Indeed, there was a four-fold enrichment of circALTO in exosomes compared to its levels in total cellular RNA (Fig 5E).

Studies have demonstrated that exosomal mRNAs and ncRNAs, including EBV derived miRNAs, can be transferred between cells [53,54]. The purified EVs were used to determine whether circALTO can be taken up by UISO cells through exosomes. UISO cells were cultured with WaGa exosomes. To do this, EVs were purified, fluorescently labeled with 1,1′-dioctadecyl-3,3,3′3′-tetramethylindocarbocyanine perchlorate (DiI), and incubated with UISO cells for 24 h to confirm the efficiency of uptake. Fluorescence was only detected in UISO cells incubated with labeled WaGa EVs, but not with PBS containing dye (Fig 5F), suggesting specific uptake of the EVs into endocytic compartments.

**Fig 6:**
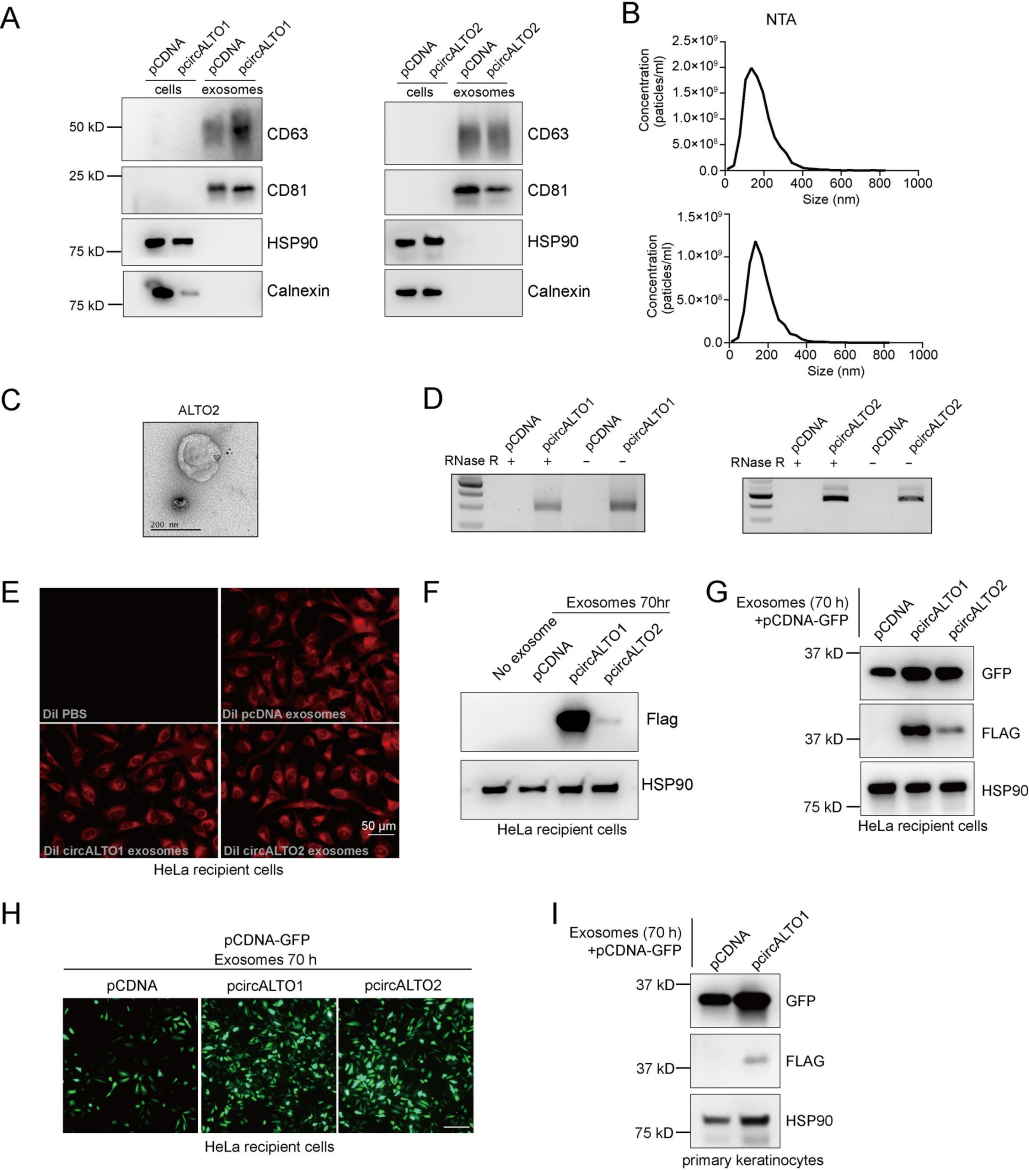
Exosomal circALTO can mediate ALTO expression in HeLa cells and keratinocytes. (A) Western blot analysis of exosomal marker in exosomes extracted from 293 T cells transfected with pCDNA vector control or Flag-circALTO1/2. CD63 and CD81 are exosomal marker proteins, while calnexin and β-tubulin exclude contamination from the cytoplasm and cellular organelles. (B) Size distribution analysis of total exosomes isolated from the Flag-circALTO1 (upper panel) and Flag-circALTO2 (bottom panel) expressed 293T cells. (C) TEM images of exosomes isolated from circALTO2 expressed 293T cells confirm expected cup-shaped morphology. (D) RT-PCR analysis of total RNA from exosomes of transfected with pCDNA vector control or Flag-circALTO1/2 293 T cells with and without RNase R treatment. (E) Fluorescence of HeLa cells incubated with DiI labeled exosomes from indicated cells by fluorescent microscopy. Scale bar = 50 μm. (F) Purified exosomes from 293T cells transfected with Flag-circALTO1/2 were transferred to HeLa cell. Expression of Flag was assessed by WB. HSP90 is the loading control. (G) The indicated purified exosomes were transferred to HeLa cell. After 70 hours, cells were transfected with pCDNA-GFP (48 hours), then lysates were generated to determine expression of Flag and GFP by WB. HSP90 is the loading control. (H) HeLa cells treated as previously described (G) were imaged by IF. Scale bar = 200 μm. (I) The indicated purified exosomes were transferred to HeLa cell. After 70 hours, cells were transfected with pCDNA-GFP (48 hours), then lysates were generated to determine expression of Flag and GFP by WB. HSP90 is the loading control.

To test whether exosomal circALTO could be transferred between cells, we first tested whether circALTO could be purified from circALTO transfected 293T cells. Indeed, EVs containing circALTOs could also be detected in 293T cells transfected with pcircALTO1 or pcircALTO2 expression constructs. The purity of EVs isolated from 293T cells was also confirmed by WB, NTA, and TEM (Figs 6A–6C and S6B). Both circALTO1 and 2 RNAs could readily be detected in purified EVs from 293T cells (Fig 6D).

These purified EVs were used to determine whether circALTO RNAs could be transferred to HeLa cells through exosomes. EVs were purified, fluorescently labeled with DiI, and incubated with HeLa cells for 24 h. Fluorescence was only detected in HeLa cells incubated with labeled vector control or circALTO1/2 EVs, but not with PBS containing dye (Fig 6E). Next, the expression of Flag-ALTO in cell lysates was determined by immunoblotting. Flag-ALTO expression was detected in HeLa recipient cells for both circALTO vectors (Fig 6F). The expression of Flag-ALTO from circALTO1 was particularly strong, perhaps due to its potential for rolling-circle translation. Next, we tested whether circALTO RNA containing exosomes could mediate transcriptional activation as has been shown for co-transfected circALTO plasmid. Thus, exosomes were purified from pCDNA3.1, pcircALTO1, or pcircALTO2 transfected cells, and incubated with HeLa cells. Those exosome treated cells were then transfected with pCDNA-GFP and the expression of GFP was monitored by WB (Fig 6G) and IF (Fig 6H). GFP expression was notably higher in the circALTO exosome-treated cells. We repeated exosome transfer experiments using primary human keratinocytes as the recipient cells. Keratinocytes were first incubated with control or pcircALTO1 exosomes (24 hr) and then transfected with a pCDNA-GFP reporter vector. Consistent with our finding from HeLa cells, we found that pre-incubation with pcircALTO1 exosomes increased GFP expression as assessed by WB and IF. Thus, circALTO has the potential to mediate its impact transcription in a paracrine manner.

### CircALTO formation is conserved in TSPyV

**Fig 7:**
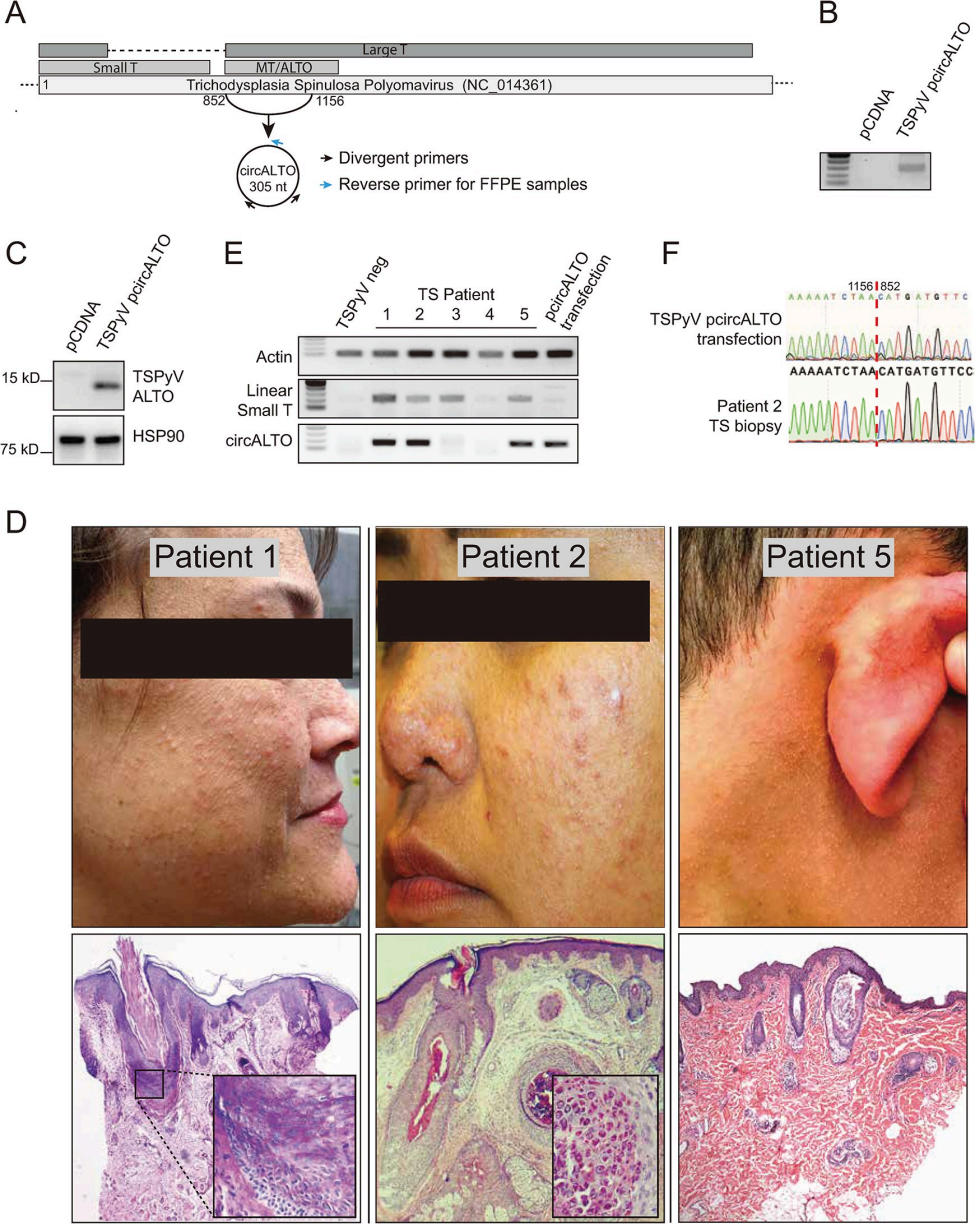
Conservation of circALTO in TSPyV. (A) Schematic representation of a predicted circALTO in TSPyV. Diagram highlights the location of the divergent primers used to detect TSPyV circALTO. (B). RT-PCR analysis of cDNA prepared from total RNA prepared with random hexamer primers. 293T cells were transfected with pCDNA vector or a construct containing the potentially backspliced early region of TSPyV. Total RNA was first treated with RNase R. (C) Western blot for TSPyV ALTO from 293T cells transfected with a pCDNA vector or the TSPyV circALTO construct. Lysates were probed with a polyclonal antibody generated against 2 peptides specific to the TSPyV ALTO, pAbMT [14]. HSP90, loading control. (D) Images from cases of TS identified in this study. Clinical images (top) from TS patients 1, 2, and 5. The photos show the characteristic folliculocentric papules on the face and neck. Histological images (bottom) show the H&E stained image of lesional skin from patients 1, 2, and 5. Affected skin shows dysmorphic hair follicles. Patient 1 inset shows expansion of inner root sheath (IRS). Patient 2 inset shows enlarged trichohyaline granules in IRS cells. (E) Total RNA was extracted from lesional biopies and cDNA prepared with random hexamer primers. RT-PCR analysis using indicated primers revealed the presence of linear small T (sT) in 4/5 TS samples and circALTO in 3/5 TS samples. A TSPyV negative squamous cell carcinoma was used as a negative control. 293T cells transfected with TSPyV circALTO was used as a positive control for circALTO. (F) Sanger sequencing of PCR products from 293T transfected cells and PCR product from patient 2 confirmed the expected backsplice junction.

Like MCPyV, TSPyV is an alphapolyomavirus whose genome has the potential to encode for ALTO [14,15,55]. The MT/ALTO genes appear to be conserved in a subset of *Alphapolyomaviruses* and the TSPyV early region contains canonical splice sites that could also be susceptible to backsplicing events. To determine whether TSPyV might also generate a circular ALTO RNA (Fig 7A), a minigene expression vector containing the ALTO-containing region of TSPyV was generated (S7A Fig). The TSPyV circALTO construct was transfected into 293T cells, and a product consistent with a circALTO product was detected by RT-PCR (Fig 7B). Like MCPyV circALTO, TSPyV circALTO was resistant to RNase R (S7B Fig). Using antibodies specific for TSPyV ALTO (pAbMT) [14], TSPyV ALTO protein could be detected in TSPyV circALTO transfected cells (Fig 7C). Thus, like MCPyV circALTOs, TSPyV circALTO is translated to ALTO in vitro. To determine whether TSPyV circALTO could be detected during TSPyV infections, we analyzed FFPE skin biopsies from five patients diagnosed with trichodysplasia spinulosa (TS). Biopsy samples were identified from patients with clinical and histological features consistent with TS (Fig 7D). All patients developed TS in the setting of immunosuppression (Table C in S7 Fig). Endpoint RT-PCR revealed that products consistent with linear early region transcripts (Linear small T) could be identified in 4 out of 5 samples, and circALTO could be detected in 3 out of 5 samples (Fig 7E). Sanger sequencing confirmed that the backsplice junction in patient tissues was identical to the product generated by transfection of the TSPyV circALTO construct (Fig 7F). Finally, we tested whether TSPyV circALTO might also promote co-transfected genes. In contrast to MCPyV ALTO, TSPyV ALTO did not promote GFP reporter expression from the CMV or EF1-α promoter as assessed by WB, IF, and qRT-PCR (S7D–S7F Fig). We conclude that circALTO formation is conserved in TSPyV; however, the function of TSPyV ALTO remains unknown.

## Discussion

EBV, KSHV, and HPV have been shown to encode for virus-derived circRNAs [25,26,28–30]. In this report, we identify and analyze polyomavirus-encoded circRNAs. We characterize both MCPyV and TSPyV-derived, protein-coding circALTO RNAs. MCPyV circALTOs can be detected in both VP-MCC lines and patient tumors. RNase R digestion and northern blotting confirm that the products are bona fide circRNAs, as opposed to RNA transcripts making multiple passes around the circular viral genome. The biological relevance of circALTO is strengthened by our identification and detection of TSPyV circALTO in TS patient samples, suggesting the evolutionary conservation of circALTO formation. While we focus only on circALTO, our analyses suggest that backsplicing in HPyV is much more widespread than previously appreciated and may involve other genes, such as late region genes with the potential to encode agnoproteins.

We provide strong evidence that circALTOs can function as templates for translation. Small interfering RNAs specifically targeting the unique backsplice (circularization) junction, but not siRNAs specific to the linear ALTO RNAs, strongly decrease ALTO protein expression in vitro. Moreover, mutation of in the MCPyV circALTO backsplicing sites suppresses expression of the ALTO protein isoform. In contrast to our findings, Abere et al were not able to find convincing evidence that circMCV-T/ALTO could be translated [56]. However, when using a mutant circMCV-T/ALTO1 that does not generate the MCPyV miRNAs, they were able to detect the circMCV-T/ALTO1 on polysomes, though ALTO expression from this construct was still not detected. Differences in circRNA expression systems, in vitro transfection conditions, and the sensitivity and specific of reagents used to detect ALTO expression may explain these discrepancies.

The protein coding capacity of circALTO in TSPyV is also conserved and was confirmed using MT/ALTO specific antibodies. Both MCPyV and TSPyV also generate a diverse array of linear transcripts that also have the potential to encode for ALTO. Our results do not exclude the possibility that these linear and other cryptic trans-spliced ALTO RNAs might contribute to some small degree of ALTO expression, as has been reported to occur for other translated circRNAs [57]. However, the observation that siRNAs specific for linear regions of ALTO do not strongly compromise ALTO expression, suggests that circular, rather than linear, ALTO RNAs are the primary ALTO-coding transcripts (Figs 3D, 3E and S4A–S4D). Though linear and circular RNAs possess different properties, it remains unclear how these multiple transcripts are differentially regulated and how they contribute differentially to viral infection.

The factors that regulate the cap-independent translation of circRNAs remain an area of active investigation. Internal ribosome entry sequences, m^6^A motifs, and novel sequences in the untranslated region (UTR) of circRNAs have all been demonstrated to promote circRNA translation [58,59]. Because the knockdown of both Mettl3/14 significantly decreased circALTO RNA levels (S4F–S4G Fig), we can not disambiguate whether m^6^A-modification is required for circRNA biogenesis, translation, or both processes. As m^6^A has been reported to play diverse roles in splicing [39], translation [38], and circularization of circRNAs [59], it is likely that the function of this abundant modification may be multifactorial and context specific.

CircALTO RNAs are perfectly complementary to the MCPyV encoded miRNA, miR-M1-5p/3p. However, because circALTO is much less abundant relative to linear ALTO mRNAs, it is unlikely that a primary function of circALTO would be to function as a decoy or sponge for miRNAs, as has been reported for other circRNAs [11,23,31,32]. Indeed, our experimental system did not suggest that circALTO could function as a miRNA sponge. While we cannot rule out some rare hypothetical conditions exist where circALTO greatly exceeds miRNA levels such that it could have sponge activity, we note that, in our miRNA repression assays, circALTO protein generated from the vector significantly enhances transcription of the luciferase reporter, while under these same conditions, no detectable sponge activity occurs (S3C and S3D Fig). Thus, the major activity of circALTO in our experimental conditions is promoting reporter gene expression rather than sponging miRNAs. Further, we find that MCPyV miRNAs actually have the potential to regulate circALTO expression. While there is clear biochemical evidence that a subset of circRNAs sponge and inhibit specific miRNAs [31,32,42], we speculate that the low abundance of most circRNAs makes it unlikely that miRNA sponging is a biologically relevant function for most circRNAs. The complex dynamics of the relationship between circALTOs, linear early region transcripts, and miRNAs during polyomavirus infections requires additional investigation.

MPyV MT enhances the transcription genes whose promoters contain binding sites for the polyomavirus enhancer A binding proteins 1 and 3 (PEA1/AP-1) and (PEA3/ETV4). These promoters are also required for MPyV replication [48,49]. In addition, SV40 large T antigen has previously been reported to promote transcription from some viral and cellular promoters [60,61]. While SV40 LT has been shown to bind multiple components of the transcriptional pre-initiation complex, the precise mechanism of its ability to promote transcription remains unclear [62]. For the first time we demonstrate a biological function for MCPyV ALTO and show that it can, like MPyV MT and SV40 LT, amplify the transcriptional activity of some promoters. Specifically, circALTO, but not splicing or ALTO start-codon mutants, enhanced the transcription of reporter genes driven by the CMV and SV40 early/late promoters. Curiously, while circALTO formation appears to be conserved, TSPyV ALTO’s ability to promote transcription, at least in the two promoters tested here, did not appear to be conserved. While we have no evidence that MCPyV or TSPyV ALTOs enhance transcription from their endogenous promoters, more thorough analyses are necessary to determine whether ALTO may impact MCPyV or TSPyV transcription.

RNA sequencing from MCPyV pcircALTO1 transfected cells suggests several pathways that might allow ALTO to impact viral pathogenesis. ALTO could synergize with sT to enhance the activation of non-canonical NF-κB signaling through NFKB2 and RELB expression, and inhibit the activation of canonical NF-κB through NFKBIA expression. Consistent with this model, analyses for potential upstream regulators of ALTO-induced gene expressed identified multiple NF-kB signaling components (S5H Fig). ALTO might also enhance viral replication by enhancing LT function through HSP70B expression. We speculate that ALTO could function as a molecular amplifier for other early region gene products, one whose expression could be finely tuned through miRNA expression. Additional studies will be necessary to identify potential interacting proteins and mechanisms for ALTO’s impact on gene expression.

Finally, we provide strong evidence that circALTO RNAs can be enriched in exosomes. While the exosome-mediated transfer and translation of linear mRNAs between cells has previously been documented, we present evidence that translated circRNAs may be transferred between cells. Consistent with the literature [36], we found circALTO to be enriched in exosomes relative to linear ALTO compared to their levels in the cell. Coupled with our confirmation that circALTO is far more stable than linear ALTO, we speculate that exosomal circALTO could be transferred to recipient cells and modulate host cell transcription in a paracrine fashion. One speculative hypothesis is that circALTO RNA-containing exosomes could promote MCPyV infection and replication in surrounding tissues by “priming” neighboring cells to be more receptive to early region gene expression and function (S8 Fig).

Exosomes also have diagnostic potential in cancer [37]. We were able to detect circALTO in WaGa cells. EVs, including exosomes, have been found in serum and variety of other bodily fluids. Thus, despite the low levels of circALTO in WaGa cells, it would nonetheless be worthwhile determining whether circALTO might have utility as a non-invasive clinical biomarker for patient with VP-MCC.

Our discovery of circALTO offers novel insights on the transcriptional complexity of polyomaviruses and extends the spectrum of viruses that encode for circRNAs. The incorporation of circRNAs into EVs, which can be transferred and expressed in other cells, raises the possibility that circALTO exerts its effects primarily in a non-cell-autonomous fashion. While MCPyV ALTO can regulate transcription, the precise role of MCPyV and TSPyV circALTO in models of infection will require further investigation. Additional studies on ALTO may yield novel therapeutic targets in HPyV infection or carcinogenesis and will broaden our understanding of the regulation of circRNA metabolism and the polyomavirus life cycle.

## Materials and methods

### Ethics statement

The UT Southwestern Institutional Review Board approved these retrospective studies (STU 072018–067). Frozen MCC tumors and normal skin were obtained from the UTSW Tissue Management Shared Resource. Excess skin biopsy tissues were obtained from patients diagnosed with trichodysplasia spinulosa by clinical and histological criteria.

### Cell culture

MKL1, MKL2, MS-1 and WaGa cells were grown in RPMI supplemented with 20% fetal bovine serum (FBS). MCC13, MCC26, and UISO cells were cultured in RPMI supplemented with 10% FBS. MCC lines were confirmed through verifying the expression of sT and assessment of cell morphology. Characteristics of these cell lines have been described previously [8, 63]. WaGa shows high expression of MCPyV early region genes [8, 46]. HeLa, HEK293 and HEK293T cells (ATCC) were cultured in DMEM and additional 10% FBS. Primary Keratinocytes (Lifeline Cell Technology) were cultured in Keratinocyte-SFSM (Life Technologies). All cells were grown at 37°C with 5% CO2.

### RNA-Sequencing (RNA-Seq) including circRNA detection

RNA-Seq was performed as previously described [46]. 293T cells (6cm plate) were transected with 1 μg of the indicated plasmid. After 48 h, total RNAs were extracted by RNeasy Miniprep Kit according to manufacturer’s instructions. The total RNA samples were then sent to Admera for RNA quality control, library preparation, and sequencing. RNA-sequencing library preparation used NEBNext Ultra II with Poly-A Selection; Illumina 2x150; 40M PE reads per sample (NEB). Trim Galore (https://www.bioinformatics.babraham.ac.uk/projects/trim_galore/) was used for quality and adapter trimming. The human reference genome sequence and gene annotation data, hg38, were downloaded from Illumina iGenomes (https://support.illumina.com/sequencing/sequencing_software/igenome.html). The qualities of RNA-sequencing libraries were estimated by mapping the reads onto mouse transcript and ribosomal RNA sequences (Ensembl release 89) using Bowtie (v2.3.4.3) [64]. STAR (v2.7.2b) [65] was employed to align the reads onto the mouse genome, SAMtools (v1.9) [66] was employed to sort the alignments, and HTSeq Python package [67] was employed to count reads per gene. DESeq2 R Bioconductor package [68, 69] was used to normalize read counts and identify differentially expressed (DE) genes. KEGG [70] pathway data was downloaded using KEGG API (https://www.kegg.jp/kegg/rest/keggapi.html) and gene ontology (GO) data was downloaded from NCBI FTP (ftp://ftp.ncbi.nlm.nih.gov/gene/DATA/gene2go.gz). The enrichment of DE genes to pathways and GOs were calculated by Fisher’s exact test in R statistical package. Upstream regulator analysis was performed with statistical measures of Ingenuity Pathway Analysis [71] and transcription factor targets from TRRUST v2 [72]. RNA-Seq available under accession number GSE171397.

Identification of circular RNA from RNA-Seq data of viruses with circular genomes was essentially described previously [30]. In brief, a custom pipeline termed vircircRNA was used (https://github.com/jiwoongbio/vircircRNA) and Burrows-Wheeler Aligner (BWA, v0.7.15) were used for aligning RNA-Seq reads. Splice reads mapped in chiastic order were defined as back-splice reads from circular RNAs. The genome sequences of MCPyV (NC_010277.2) were downloaded from National Center for Biotechnology Information (NCBI) database. We searched public sequencing data of MCPyV from NCBI Sequence Read Archive (SRA). Backsplice junctions were detected from the following SRA samples: ERS760222-5 [73].

### End-point PCR, qRT-PCR and RNase R treatment

End point PCR and quantitative real-time RT-PCR (qRT-PCR) for circRNAs were performed as previously described [30]. In brief, total RNA was extracted from cells using the RNeasy Mini Kit (Qiagen, 74104). 2 μg of total RNA was subjected to RNase R treatment [30] with 5U RNase R (Lucigen, RNR07250) at 37°C for 30 min and then placed on ice. Water was used in mock reactions. After incubating the sample with 1 μl of 1mM EDTA, 1 μl random hexamer (100 μM) and 1 μl of 10 mm dNTPs at 65°C for 5 min, the denatured RNA was used for the cDNA synthesis with a Superscript IV RT system (ThermoFisher, 18091050) according to manufacturer’s instructions. For GFP qRT-PCR, total RNA was extracted from cells using the RNeasy Mini Kit, and the genomic DNA was extracted from cells using Quick Extract DNA Extraction Solution (ThermoFisher, NC9904870) according to the instructions. The total RNA was used for the cDNA synthesis with iScript cDNA Synthesis Kit (Biorad, 1708891) according to manufacturer’s instructions. End point PCR was performed with SapphireAmp (Takara, RR350B). Cycling conditions for circRNA were as follows: 95°C 5 min, followed by 40 cycles of 95°C 1 min, 62°C 1 min, 72°C 2 min, and a final elongation step at 72°C for 10 min. Cycling conditions for linear mRNA is the same condition with 25 cycles. For end point PCR, 2 μl cDNA templates were used for both circular and linear PCR reactions. For qRT-PCR, cDNA products and DNA products were diluted to 1:10 and 2 μL was used as template for real time PCR reaction with PowerUp SYBR Green (Applied Biosystems, A25779). The primers used for both end point PCR and qRT-PCR analysis are listed in S1 Table.

### Northern blots

Northern blotting for circular RNA analyses was performed as previously described [30]. Briefly, total RNAs were extracted using the TRIzol reagent (Invitrogen). 20–30 μg of total RNA from cancer cell lines was treated with RNase R or 8 μg of total RNA used for mock treatment. RNA samples then separated on 1.5–2.0% formaldehyde agarose gels in MOPS buffer. The RNA was transferred to the Hybond-N+ membrane (GE Lifesciences) with 10xSSC. RNA hybridization was carried out in PerfectHyb buffer (Sigma) overnight at 65°C. Probes were produced by PCR amplification in the presence of [α-^32^P] dCTP. Primers used for generating the probes are listed in S1 Table.

### Actinomycin D treatment

WaGa suspension cells were grown in six-well plates. cells were incubated with 2 μg/ml Actinomycin D (Sigma-Aldrich) or DMSO as a control and collected at indicated time points.

2 μg total RNA was subjected to 5 U RNase R treatment at 37°C for 30 min. After the cDNA synthesis, the transcript level of circALTO and ALTO mRNA were analyzed by qRT-PCR.

After 293T cells were grown overnight in six-well plates, cells were transfected with pCDNA-GFP reporter vector (1ug) and indicated plasmids including pCDNA control vector (1 ug), Flag-circALTO1 (1 ug) and Flag-circALTO2 (1 ug) plasmids. After 48 hours, cells were incubated with 2 μg/ml Actinomycin and collected at indicated time points.

### Nuclear and cytoplasmic fractionation

Fractionation was performed according to the protocol performed before [30]. Typically, cells grown on 35mm dishes were used as the starting material. After being trypsinized and pelleted, cell were resuspended in 250 μl of ice-cold Buffer I [0.5% Triton X-100, 0.32M sucrose, 3mM CaCl2, 2mM MgCl2, 0.1mM EDTA, 10mM Tris (pH 8.0), 50mM NaCl, 1mM DTT, 0.04 U/μl RNase inhibitor (ThermoFisher, 18091050)]. After a 15 min incubation on ice, cells were centrifuged at 500 g for 5 min at 4°C. The supernatant was collected for the cytoplasmic fraction and the pellet was resuspended in 250 μl of Buffer I for the nuclear fraction. Afterward soluble fraction RNA was then extracted using RNeasy Mini Kit (Qiagen, 74104) and cDNA synthesis with Superscript IV RT system (ThermoFisher, 18091050) using random hexamers. The transcript levels were examined using gene specific primers by qRT-PCR.

### Luciferase reporter assay

The luciferase reporter assay performed as described before [11]. In brief, 293 or 293T cells in 12-well plates were transfected with 5–7.5ng of the pCDNA3.1dsRlucMCVTAg plasmid, as well as 5ng of pCDNA3.1dsFFluc as a transfection control, along with 1 ug of the indicated plasmids per well (pCDNA vector 1ug, pCDNA vector 800ng + MCPyV miRNA 200ng, MCPyV miRNA 200ng + circALTO1 800ng, MCPyV miRNA 200ng + circALTO2 800ng). After 48 h transfection, cells were harvested and analyzed using a dual luciferase assay system (Dual-Glo luciferase assay system; catalog no. E2980) according to manufacturer’s instructions (Promega). Results are presented with the *Renilla* luciferase levels normalized by the firefly luciferase levels. To test for transcriptional activation, 293 cells in 12-well plates were transfected with 5 ng of the pCDNA3.1dsRlucMCVTAg and pCDNA3.1dsFFluc plasmid along with 1 ug of either pCDNA vector, circALTO1, or ALTO1-SASD. Luciferase expression was analyzed 48 hr post-transfection.

### Constructs and oligonucleotide transfection

Wild type circALTO and mutant circALTO promoter fragments were generated by gene synthesis (IDT or GeneWiz) based on the reference sequences for MCPyV (NC_010277.2) and TSPyV (NC_014361.1). Exact sequences listed in S1 Table. Gene synthesis fragments were PCR amplified (Q5 High-Fidelity 2X Master Mix, New England BioLabs) and cloned into pCDNA3.1 after restriction digest with the appropriate enzymes. The pU-5864 reporter plasmid contains the EF1-α promoter and 5’ untranslated region in addition to the upstream regulatory region from BPV1 [74]. Additional promoter sequences were generated by gene synthesis (IDT), PCR amplified, and cloned into pU-5864 after restriction digest with *Mlu*I and *Eco*RI. The ALTOw plasmid, containing a codon-modified ALTO ORF, was cut by *Kpn*I and *Not*I enzyme, blunted with Klenow and circularized to remove a GFP reporter (Klenow, New England BioLabs). All constructs were confirmed by Sanger Sequencing. Constructs were transiently transfected into 293T cells with Lipofectamine 3000 reagents (ThermoFisher) according to manufacturer’s instructions. After 48–72 h transfection, cells were harvested for fractionation, RNA preparation, and WB. All siRNAs were synthesized by Sigma-Aldrich. The 293T cells co-transfected with 80 pmol of the indicated siRNA and 1 μg of the indicated constructs using Lipofectamine 3000 without P3000 reagent. After 68–72 h transfection, cells were harvested for RNA preparation, WB, and m^6^A IP. Detailed information on sequences used in this study are listed in S1 Table and https://ccrod.cancer.gov/confluence/display/LCOTF/Support.

### m^6^A RNA immunoprecipitation assay

Total RNA was harvested 48–72 h post-transfection and extracted by (Qiagen, 74104). 5 μg total RNA was incubated with 1 μg m^6^A polyclonal antibody (Synaptic Systems# 202003) [75] and beads with rotation at 4°C. Immunoprecipitated RNA was then purified using QIAgen RNeasy columns. Purified immunoprecipitated RNA, along with 10% input RNA were then reverse transcribed by Superscript IV RT system (ThermoFisher, 18091050) using random hexamers. The modification levels were examined using gene specific primers by qRT-PCR. Percentages of m^6^A modified RNA for both circALTO and SON were calculated based on the input amount.

### Western blotting

Whole cells were trypsinized and harvest subjected to Laemmli sampling buffer and boiled for at 95°C for 10 min. The protein extracts were separated on SDS-PAGE gels, transferred to PVDF membranes and incubated with indicated antibodies. The antibodies were purchased from commercial sources: anti-FLAG HRP (1:1000, Sigma, A8592), anti-HSP90 (1:1000, Cell Signaling Technologies, 4877S), anti-CD63 (1:600, BD Biosciences, 556019), anti-CD81 (1:600, R&D Systems, MAB4615), anti-Calnexin (1:1000, Cell Signaling Technologies, 2679T), anti-β-Tublin (1:1000, Proteintech, 10094-1-AP), anti-Mettl3 (1:1000, Proteintech, 15073-1-AP), anti-GFP (1:500, Santa Cruz Biotechnology, sc-9996), ALTO antiserum [13] (1:5000), and TSPyV pAbMT (1:100) [14]. Membranes were then incubated with the appropriate HRP-conjugated secondary antibody (1:000, anti-mouse IgG, Cell Signaling Technologies, 7076P2; 1:1000, anti-rabbit IgG, ThermoFisher, G21234) and developed with an ECL system (Perkin Elmer, NEL104001).

### Immunofluorescence

The 293T cells were plated into Nunc 4-well chamber slides (ThermoFisher 154453) and transfected with indicated plasmids and siRNA. After 48 h transfection, cells were fixed in 4% paraformaldehyde at RT for 10 min, followed by 0.1% Triton X-100 permeabilization at RT for 10 min. Cells were then incubated with FLAG antibody (1:100, Sigma, F1804) overnight, then stained with Alexa 488-conjugated secondary antibody (ThermoFisher). Samples were then subjected to DAPI nuclear counterstain (Vector Labs), and imaged by fluorescent microscope.

### Total exosome isolation and transfer

Exosome isolation was performed according to a published protocol [76]. Cell-conditioned medium was collected grown for 3 days in cell culture flasks (WaGa cells) or 150 mm plates (MCC26, pCDNA and Flag-circALTO1/2 transfected 293T cells) with exosome-depleted FBS (Invitrogen). The collected media was first centrifuged at 300×g for 10 min at room temperature to pellet and remove cells. The following centrifugation steps were performed at 4°C. In brief, the supernatant was spun at 2,000×g for 15 min. Then, the supernatant was centrifuged at 10,000×g for 60 min. Next, the media supernatant was passed through a 0.22 μm pore PES filter (Millipore). This supernatant was ultracentrifuged at 100,000×g for 90 min in a SW-28Ti Rotor Swinging Bucket rotor (Beckman Coulter) and resuspended in PBS, then centrifuged at 100,000×g for 90 min again. The resulting Exosomes pellet resuspended in 250 μL of PBS and stored at −80°C for further use. Exosomes were then subjected to RNA isolation, WB, and TEM. The RNA isolated form exosome using Total Exosome RNA & Protein Isolation Kit (Invitrogen). For western blotting, the exosomes in PBS directly subjected to Laemmli sampling buffer with 2-mercaptoethanol or without 2-mercaptoethanol (CD63 and CD81) and boiled at 95°C for 10 min.

### Transmission electron microscopy (TEM)

Exosome samples were adsorbed to glow-discharged carbon-coated 400-mesh copper grids for 3 minutes and then stained with 2% (weight/volume) uranyl acetate in water, rinsed briefly with water and air dried. The grids were visualized on a FEI Tecnai 12 TEM at 80 kV. Images were captured on a Gatan Orius CCD camera with Gatan Digital Micrograph software.

### Nanoparticle tracking analysis (NTA)

Samples were diluted 1:100 for nanoparticle tracking analysis with the ZetaVew particle analyzer (Particle Metrix). The size (nm) and concentration (cm^-3^) was used to determine the extracellular vesicle size using GraphPad Prism.

### Transfer and DiI-labeled exosomes to HeLa cells

HeLa cell were planted in 24 well plates and grew until 70% confluence to use. Cells washed twice with PBS and incubated with 100 μl indicated exosomes for 70 hours at 37°C in exosomal-depleted FBS (Invitrogen) supplemented media. Whole cells were washed twice with PBS, trypsinized and harvest subjected to Laemmli sampling buffer with 2-mercaptoethanol and boiled for at 95°C for 10 min. 1 μM DiI (Invitrogen) labeled with purified exosomes as previously described [77]. Unbound DiI were removed by Exosome Spin Columns (Invitrogen). Purified exosomes were added to HeLa cells and incubated for 24 h. Recipient cells were then washed in PBS, fixed in 4% paraformaldehyde, and imaged by fluorescent microscope. For GFP transcript assay, 100 μl purified exosomes were added to HeLa cells in 24-well plates and incubated for 24 h. Next, cells were transfected with pCDNA-GFP reporter vector, and after 46 hours, cells were collected for experiments (70 h total incubation with exosomes). For primary keratinocytes, 200 μl purified exosomes were added to the cells in 12-well plates and incubated for 24 h, then cells were transfected with pCDNA-GFP reporter vector using the TransIT-Keratinocyte Transfection Reagent (Mirus Bio) and incubated for 46 h before collections for assays.

## Supporting information

S1 Fig Identification of MCPyV circRNAs. (A) Table of MCPyV circRNAs identified in SRA datasets containing location of putative circRNA, read count, and backsplice ratio. (B) Sanger sequencing of the non-specific band between the circALTO1 and circALTO2 products from WaGa cells show the inclusion of non-MCPyV sequence at the backsplice junction. (C) Endpoint RT-PCR analysis of circALTOs from VP-MCC lines (MKL-1, MKL-2, MS1 and WaGa) and VN-MCC (MCC13, MCC26 and UISO). Total RNA treated with RNase R. (D) Pathological features of the patient MCC samples. (E) Sanger sequencing of PCR products from the sample No. 28548 confirmed the expected backsplice junction for circALTO2.
(TIF)

S2 Fig Characterization of circALTO constructs. (A) Schematic diagram of circALTO1 (Left panel) and circALTO2 (Right panel) expression constructs generated in vitro. The location of QKI sites, ORF, 3xFLAG epitope-tag (present in ‘FLAG’), mutated splice sites (short for ‘SASD’), and siRNAs used in subsequent knockdown assay were indicated in the diagram. (B) The formation of circALTOs from 293T cells co-transfected with pcDNA3.1-circALTO1/2 constructs were confirmed by RT-PCR (Left panel). Sanger sequencing of PCR products showed the non-specific backsplice junction with the insertion of additional nucleotides (Right panel). (C) Readthrough transcript from 293T cells co-transfected with pcDNA3.1-circALTO1/2 constructs with or without RNase R treatment were analyzed by RT-PCR. Primers designed from CMV promoter region to the regions that flanked circALTOs. (D) Northern blot of total RNA from 293T cells co-transfected with vector control and pcDNA3.1-circALTO2 constructs probed with linear ALTO after RNase R treatment. Arrows indicates RNase R resistant band circALTO2. (E) Nuclear and cytoplasmic fractionation assay of 293 T cells co-transfected with circALTO1 construct performed by qRT-PCR. MALAT1 and *ACTB* served as fractionation controls. Values normalized to the enriched fraction. Error bars represent the SD of three biological replicates.
(TIF)

S3 Fig CircALTO functions as a transcriptional inducer but not a miRNA sponge in vitro. (A) 293T cells were transfected with Renilla luciferase reporter with MCPyV miRNA (MCV350) and the indicated plasmids including pcDNA3.1 control vector, pCDNA circALTO1 or pCDNA circALTO2 expression plasmid. Firefly luciferase served as a transfection control and Renilla luciferase levels are plotted normalized relative to firefly luciferase levels (n = 2 biological replicates). (B) 293 cells were transfected with Renilla luciferase reporter with MCPyV miRNA (MCV339) and the indicated plasmids. n = 2 biological replicates. (C) circALTO1 enhanced reporter gene expression irrespective of MCPyV miRNA activity. Under conditions where Rluc is being targeted by MCPyV miRNA, this reporter and the non-miRNA-targeted FF-Luc both display enhanced expression in the presence of circALTO. (D) Under conditions where expression of circALTO1 enhanced reporter gene expression, it does not alleviate MCPyV-miRNA-mediated repression.
(TIF)

S4 Fig Characterization of circALTOs with siRNAs. (A) qRT-PCR analysis for circALTO and ALTO mRNA in co-transfected Flag-circALTO1 constructs 293T cells treated with the indicated siRNAs (see S3A Fig). *ACTB* served as the internal control. Error bars represent the SD (n = 3 biological replicates). (B) Western blot for FLAG from 293T cells co-transfected with Flag-circALTO1 and the indicated siRNA. HSP90 serves as loading control. (C) qRT–PCR analysis for circALTO and ALTO mRNA in co-transfected Flag-circALTO2 constructs 293 T cells with siRNAs (see S3A Fig). *ACTB* served as the internal control. Error bars represent the SD (n = 3 biological replicates). (D) Western blot for FLAG from 293 T cells co-transfected with Flag-circALTO2. HSP90 is the loading control. (E) 293T cells were transfected with the Flag-circALTO1/2 plasmids alone and Flag-circALTO1/2 with indicated siRNAs. After 48 h of transfection, the cells were fixed and stained for FLAG (green), and DAPI (blue). Scale bar = 50 μm. (F) Western blots for METTL3 and Flag from 293T co-transfected with control or METTL3/14 siRNA and circALTO1 construct. HSP90, loading control. (G) qRT-PCR of circALTO of 293T co-transfected with control or METTL3/14 siRNAs and circALTO1/2 construct. Error bars represent the SD (n = 3 biological replicates). The *P* value was determined by unpaired, two-tailed t-test, *<0.05, **<0.01.
(TIF)

S5 Fig Analysis of MCPyV ALTO transcriptional regulation. (A) A construct in which all potential ATG start codons have been mutated, circALTO1-ΔATG, was generated. Endpoint RT-PCR analysis for pcircALTO of total RNA prepared from 293T cells transfected with pCDNA, pcircALTO1, and pcircALTO1-ΔATG indicate that both pcircALTO1 constructs are efficiently circularized. (B) 293T cells were co-transfected with pCDNA-GFP plasmid and either pCDNA empty control, pcircALTO1, or pcircALTO2. DNA was prepared from transfected cells and amounts of GFP plasmid were assessed by qPCR. (C) 293T cells were co-transfected with pCDNA-GFP and pCDNA control vector or circALTO1. After 48 hours, actinomycin D added to the transfected cells. GFP transcript levels in the presence of Actinomycin D at the indicated time points were assessed by qRT–PCR analysis. Error bar = SD from one biological replicate. Results are representative of 2 independent experiments. GFP levels were first normalized to 18S and then normalized to levels at the pre-treatment (0 h) time point. (D) Western blotting of 293T cells co-transfected with plasmids expressing GFP under control of PGK or EF1-a promoter together with the indicated FLAG-tagged circALTO construct. HSP90 served as a loading control. (E) This table summarizes pcircALTO1 mediated transcriptional induction of GFP by the indicated promoter when normalized to the pCDNA vector control as measured by qRT-PCR of GFP transcripts from co-transfected 293T cells. Bold italics indicates promoters whose expression was significantly induced by pcircALTO1 (P value <0.01 as determined by unpaired, two-tailed t-test). (F) Western blotting of cells co-transfected with pCDNA-GFP (CMV promoter) and the indicated ALTO expression plasmids (or pCDNA control plasmid). (G) Heatmap of genes significantly overexpressed in circALTO1 expressing cell compared to vector control (log2 FC>1.5, adjusted p<0.001). (H) Upstream regulator analysis identifies transcriptional regulators shared by multiple induced genes.
(TIF)

S6 Fig Characterization of exosomes from MCC26 and 293T cells co-transfected with pCDNA vector. (A-B) Size distribution analysis of total exosomes isolated from MCC26 cells (A) and 293T cells co-transfected with pCDNA vector (B).
(TIF)

S7 Fig Identification and characterization of TSPyV circALTO. (A) Schematic diagram of TSPyV circALTO expression constructs generated in vitro. The location of QKI sites, ORF, and splice sites were indicated in the diagram. (B) RT-PCR analysis of total RNA from 293T cells co-transfected with MCPyV circALTO or TSPyV circALTO plasmids with and without RNase R treatment. (C) Table summarizing the clinical characteristics and RT-PCR results of TS patients utilized in this study. Only patient 1 has previously been reported. (D) Western blot for GFP and ALTO from 293T cells were co-transfected with the indicated plasmids: either pU-5864 or pCDNA-GFP AND either pCDNA control vector or TSPyV circALTO plasmids. HSP90 serves as the loading control. (E) IF images of 293T cells co-transfected with either pU-5864 or pCDNA-GFP and either pCDNA vector or TSPyV pcircALTO for 48 hours. Scale bar = 200 μm. (F) qRT-PCR of GFP transcripts from 293T cells co-transfected with either pU-5864 or pCDNA-GFP AND either pCDNA or TSPyV circALTO. ACTB served as the internal control. Error bars = SD from a single experiment. Results are representative of 2 independent experiments.
(TIF)

S8 Fig Graphical Abstract. Speculative model for the regulation and function of MCPyV circALTO.
(TIF)

S1 Table Sequences used in the study. (XLSX)
