# Supplementary figures and images for: Characterization of ALTO-encoding circular RNAs expressed by Merkel cell polyomavirus and trichodysplasia spinulosa polyomavirus
Source: PLoS Pathog. 2021 May 17;17(5):e1009582. doi: 10.1371/journal.ppat.1009582 (PMC8158866; doi:10.1371/journal.ppat.1009582)

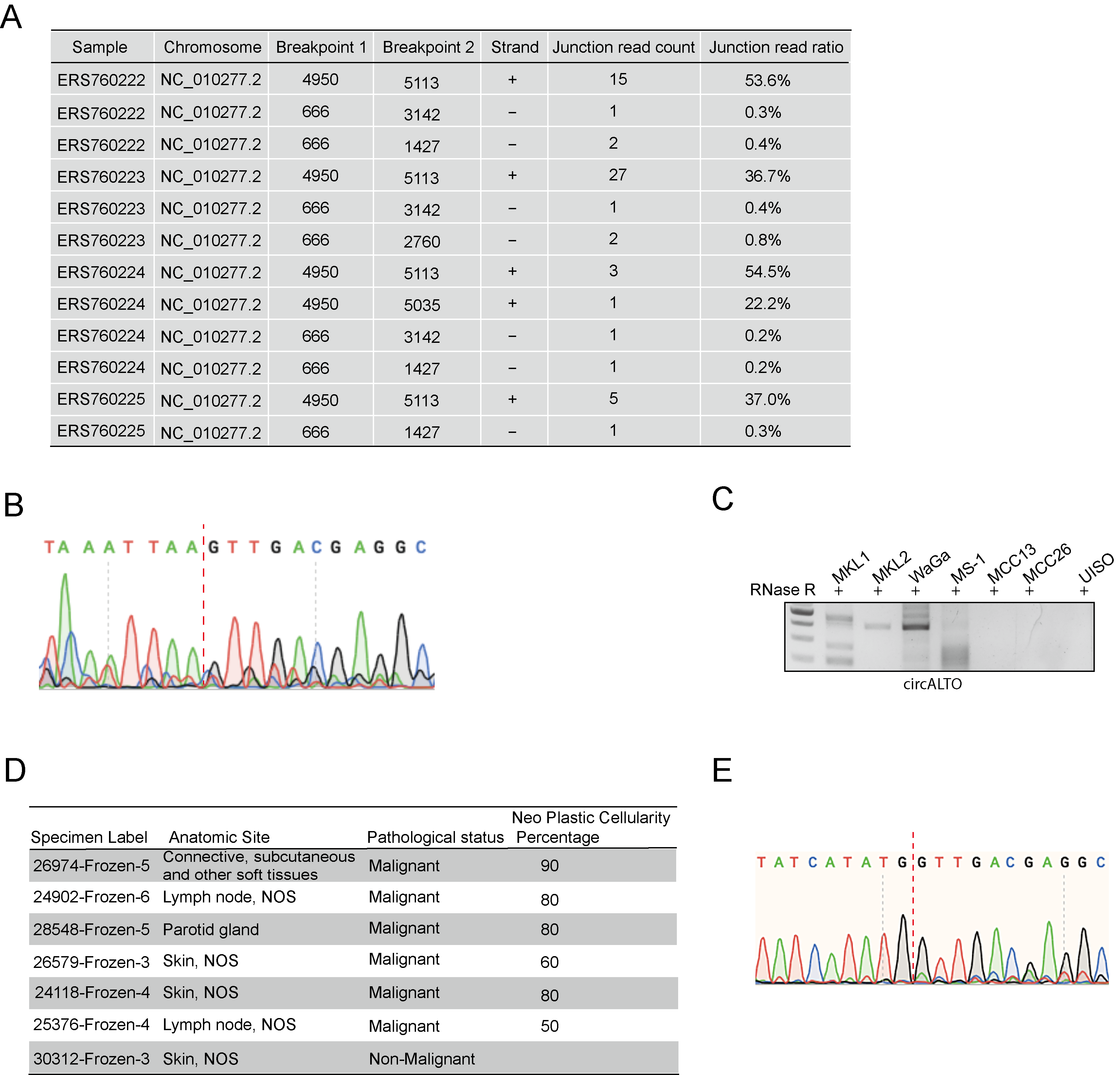

Supplement: S1 Fig — (A) Table of MCPyV circRNAs identified in SRA datasets containing location of putative circRNA, read count, and backsplice ratio. (B) Sanger sequencing of the non-specific band between the circALTO1 and circALTO2 products from WaGa cells show the inclusion of non-MCPyV sequence at the backsplice junction. (C) Endpoint RT-PCR analysis of circALTOs from VP-MCC lines (MKL-1, MKL-2, MS1 and WaGa) and VN-MCC (MCC13, MCC26 and UISO). Total RNA treated with RNase R. (D) Pathological features of the patient MCC samples. (E) Sanger sequencing of PCR products from the sample No. 28548 confirmed the expected backsplice junction for circALTO2. (TIF) [file ppat.1009582.s001.tif]

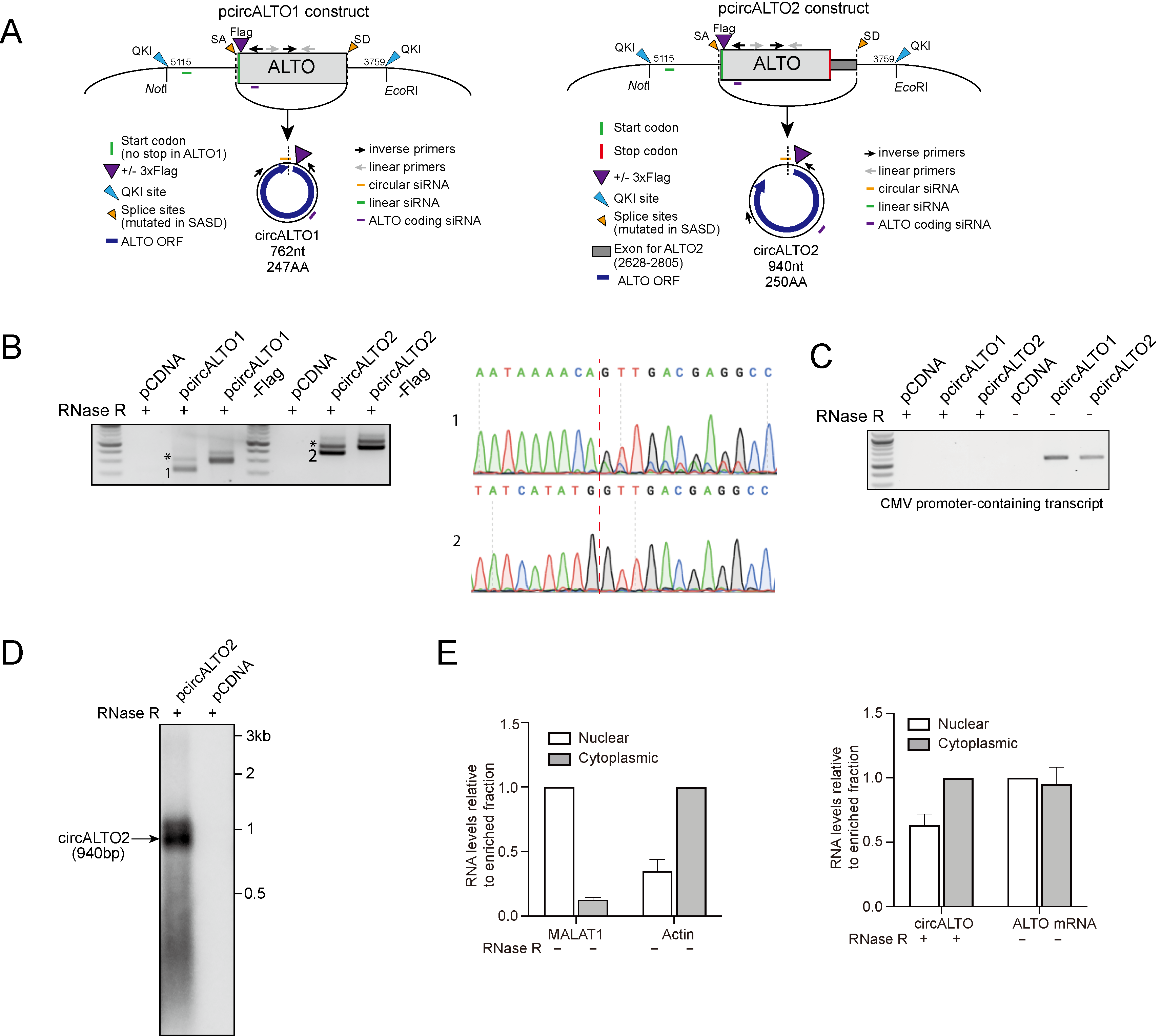

Supplement: S2 Fig — (A) Schematic diagram of circALTO1 (Left panel) and circALTO2 (Right panel) expression constructs generated in vitro. The location of QKI sites, ORF, 3xFLAG epitope-tag (present in ‘FLAG’), mutated splice sites (short for ‘SASD’), and siRNAs used in subsequent knockdown assay were indicated in the diagram. (B) The formation of circALTOs from 293T cells co-transfected with pcDNA3.1-circALTO1/2 constructs were confirmed by RT-PCR (Left panel). Sanger sequencing of PCR products showed the non-specific backsplice junction with the insertion of additional nucleotides (Right panel). (C) Readthrough transcript from 293T cells co-transfected with pcDNA3.1-circALTO1/2 constructs with or without RNase R treatment were analyzed by RT-PCR. Primers designed from CMV promoter region to the regions that flanked circALTOs. (D) Northern blot of total RNA from 293T cells co-transfected with vector control and pcDNA3.1-circALTO2 constructs probed with linear ALTO after RNase R treatment. Arrows indicates RNase R resistant band circALTO2. (E) Nuclear and cytoplasmic fractionation assay of 293 T cells co-transfected with circALTO1 construct performed by qRT-PCR. MALAT1 and ACTB served as fractionation controls. Values normalized to the enriched fraction. Error bars represent the SD of three biological replicates. (TIF) [file ppat.1009582.s002.tif]

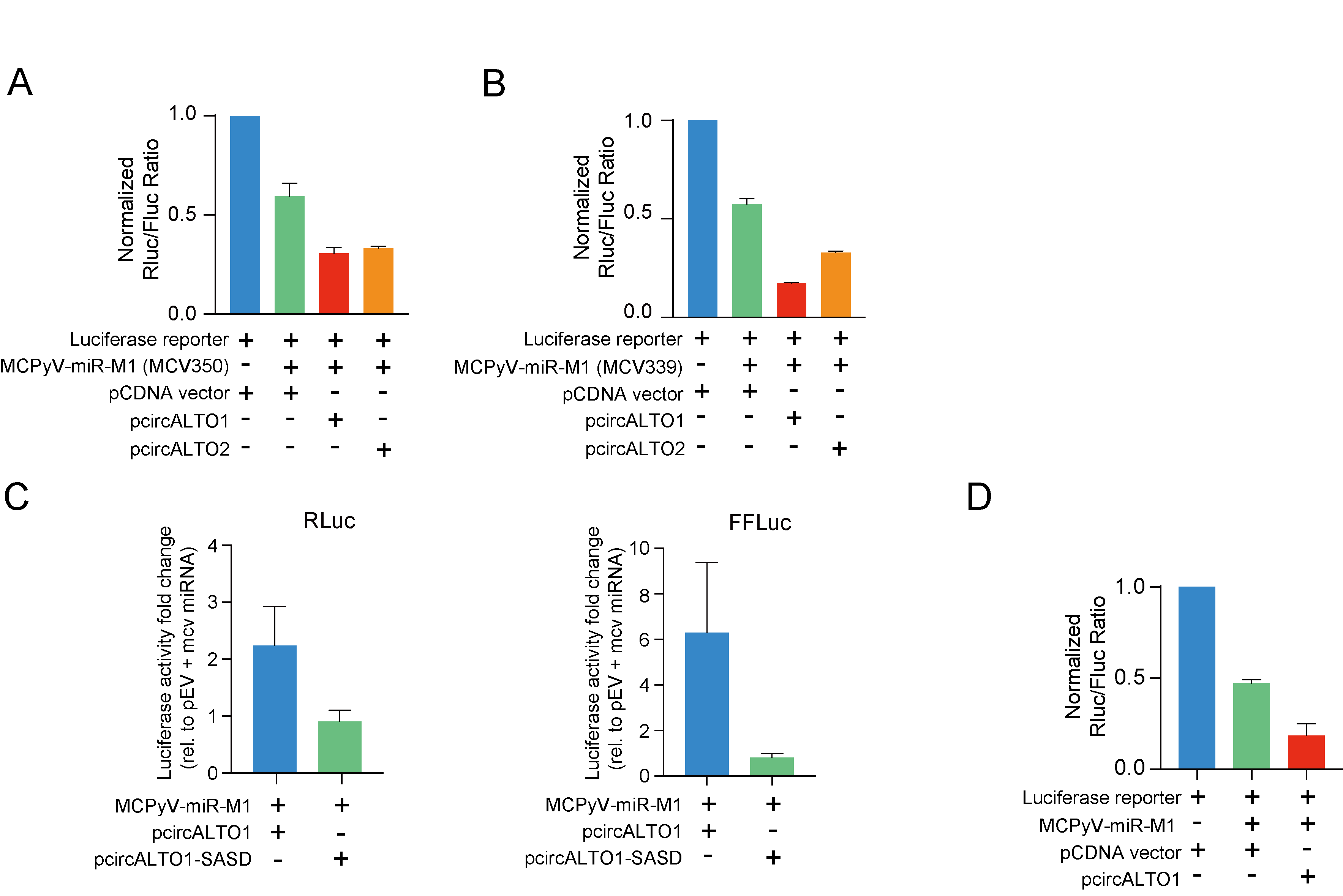

Supplement: S3 Fig — (A) 293T cells were transfected with Renilla luciferase reporter with MCPyV miRNA (MCV350) and the indicated plasmids including pcDNA3.1 control vector, pCDNA circALTO1 or pCDNA circALTO2 expression plasmid. Firefly luciferase served as a transfection control and Renilla luciferase levels are plotted normalized relative to firefly luciferase levels (n = 2 biological replicates). (B) 293 cells were transfected with Renilla luciferase reporter with MCPyV miRNA (MCV339) and the indicated plasmids. n = 2 biological replicates. (C) circALTO1 enhanced reporter gene expression irrespective of MCPyV miRNA activity. Under conditions where Rluc is being targeted by MCPyV miRNA, this reporter and the non-miRNA-targeted FF-Luc both display enhanced expression in the presence of circALTO. (D) Under conditions where expression of circALTO1 enhanced reporter gene expression, it does not alleviate MCPyV-miRNA-mediated repression. (TIF) [file ppat.1009582.s003.tif]

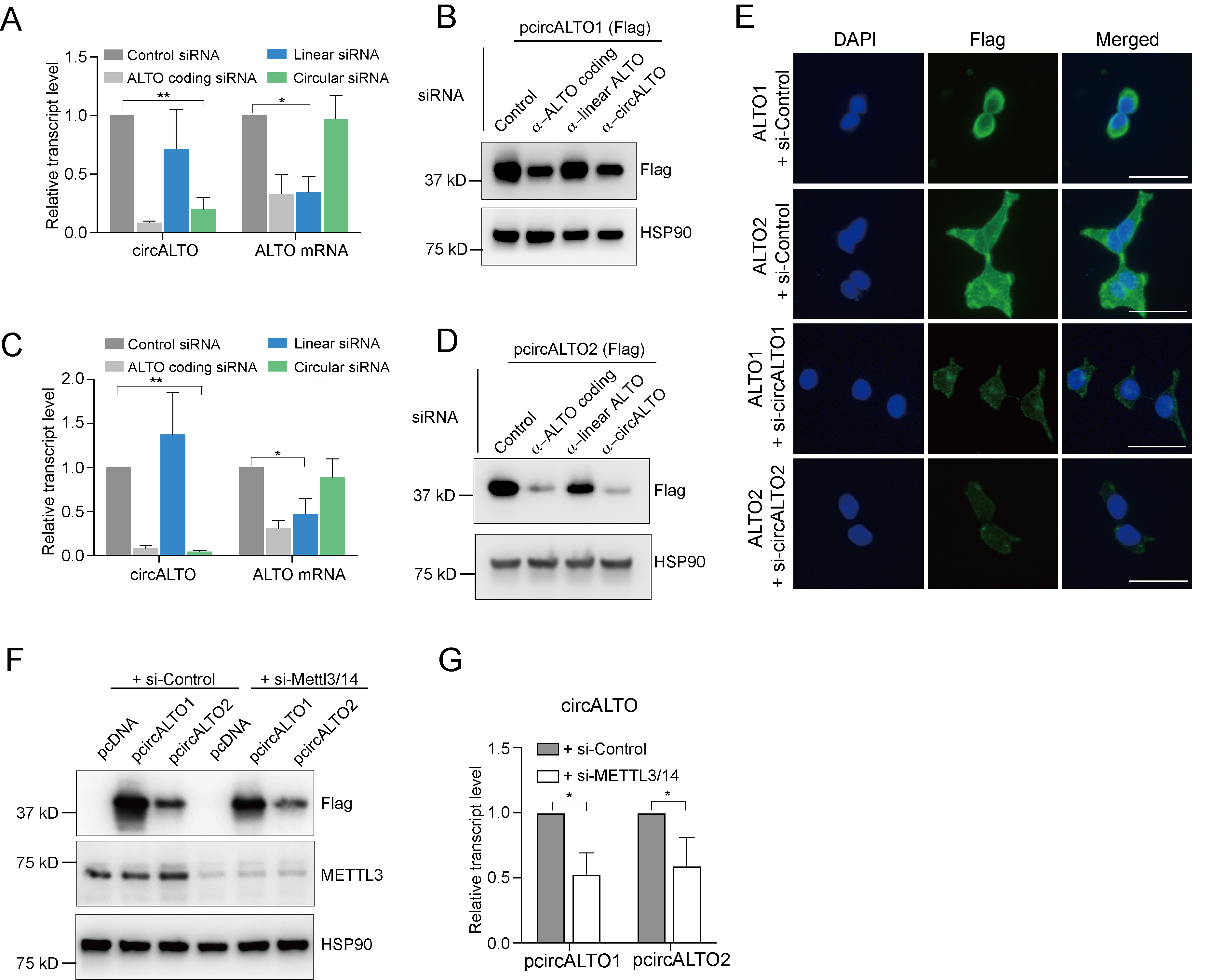

Supplement: S4 Fig — (A) qRT-PCR analysis for circALTO and ALTO mRNA in co-transfected Flag-circALTO1 constructs 293T cells treated with the indicated siRNAs (see S3A Fig). ACTB served as the internal control. Error bars represent the SD (n = 3 biological replicates). (B) Western blot for FLAG from 293T cells co-transfected with Flag-circALTO1 and the indicated siRNA. HSP90 serves as loading control. (C) qRT–PCR analysis for circALTO and ALTO mRNA in co-transfected Flag-circALTO2 constructs 293 T cells with siRNAs (see S3A Fig). ACTB served as the internal control. Error bars represent the SD (n = 3 biological replicates). (D) Western blot for FLAG from 293 T cells co-transfected with Flag-circALTO2. HSP90 is the loading control. (E) 293T cells were transfected with the Flag-circALTO1/2 plasmids alone and Flag-circALTO1/2 with indicated siRNAs. After 48 h of transfection, the cells were fixed and stained for FLAG (green), and DAPI (blue). Scale bar = 50 μm. (F) Western blots for METTL3 and Flag from 293T co-transfected with control or METTL3/14 siRNA and circALTO1 construct. HSP90, loading control. (G) qRT-PCR of circALTO of 293T co-transfected with control or METTL3/14 siRNAs and circALTO1/2 construct. Error bars represent the SD (n = 3 biological replicates). The P value was determined by unpaired, two-tailed t-test, *<0.05, **<0.01. (TIF) [file ppat.1009582.s004.tif]

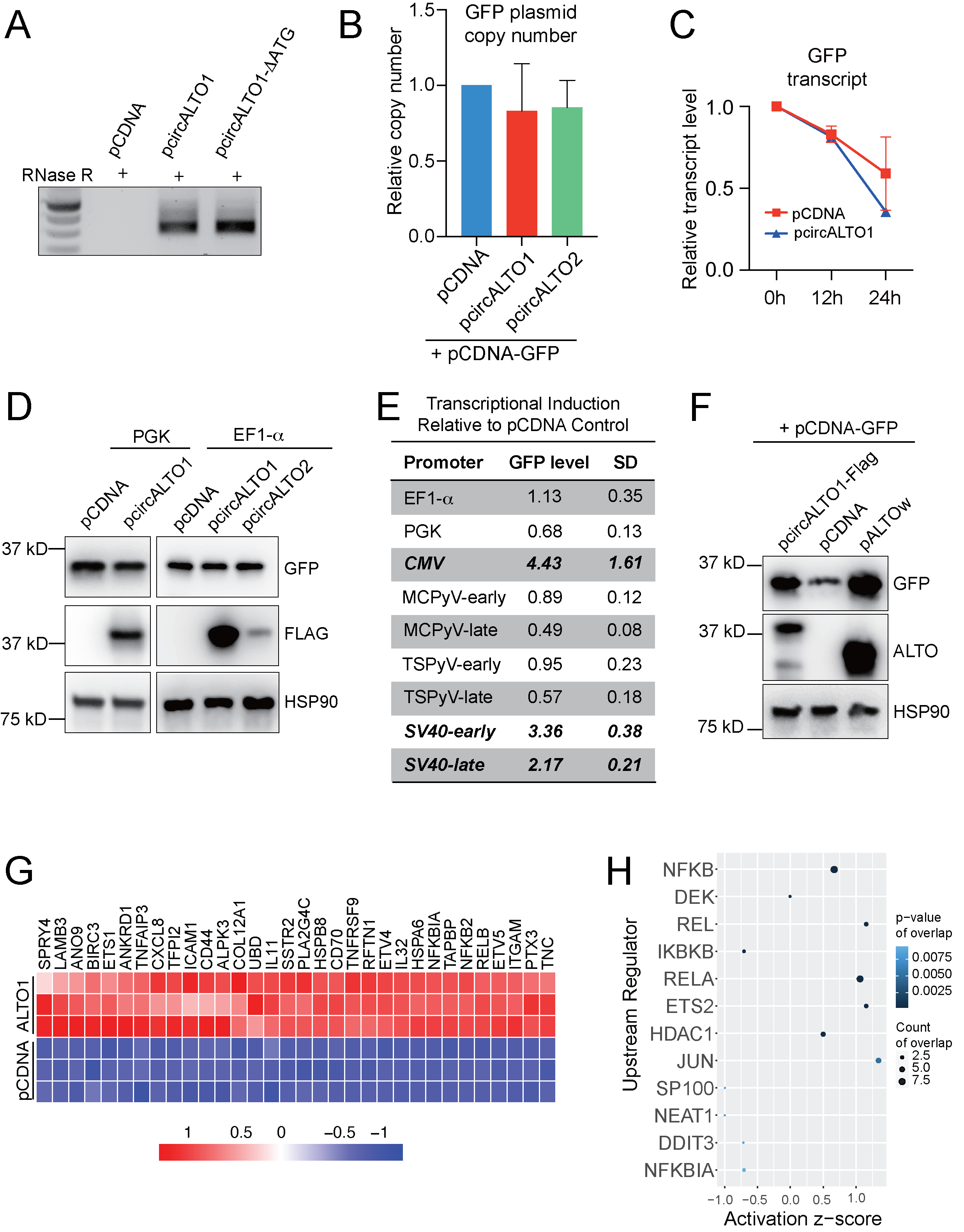

Supplement: S5 Fig — (A) A construct in which all potential ATG start codons have been mutated, circALTO1-ΔATG, was generated. Endpoint RT-PCR analysis for pcircALTO of total RNA prepared from 293T cells transfected with pCDNA, pcircALTO1, and pcircALTO1-ΔATG indicate that both pcircALTO1 constructs are efficiently circularized. (B) 293T cells were co-transfected with pCDNA-GFP plasmid and either pCDNA empty control, pcircALTO1, or pcircALTO2. DNA was prepared from transfected cells and amounts of GFP plasmid were assessed by qPCR. (C) 293T cells were co-transfected with pCDNA-GFP and pCDNA control vector or circALTO1. After 48 hours, actinomycin D added to the transfected cells. GFP transcript levels in the presence of Actinomycin D at the indicated time points were assessed by qRT–PCR analysis. Error bar = SD from one biological replicate. Results are representative of 2 independent experiments. GFP levels were first normalized to 18S and then normalized to levels at the pre-treatment (0 h) time point. (D) Western blotting of 293T cells co-transfected with plasmids expressing GFP under control of PGK or EF1-a promoter together with the indicated FLAG-tagged circALTO construct. HSP90 served as a loading control. (E) This table summarizes pcircALTO1 mediated transcriptional induction of GFP by the indicated promoter when normalized to the pCDNA vector control as measured by qRT-PCR of GFP transcripts from co-transfected 293T cells. Bold italics indicates promoters whose expression was significantly induced by pcircALTO1 (P value <0.01 as determined by unpaired, two-tailed t-test). (F) Western blotting of cells co-transfected with pCDNA-GFP (CMV promoter) and the indicated ALTO expression plasmids (or pCDNA control plasmid). (G) Heatmap of genes significantly overexpressed in circALTO1 expressing cell compared to vector control (log2 FC>1.5, adjusted p<0.001). (H) Upstream regulator analysis identifies transcriptional regulators shared by multiple induced genes. (TIF) [file ppat.1009582.s005.tif]

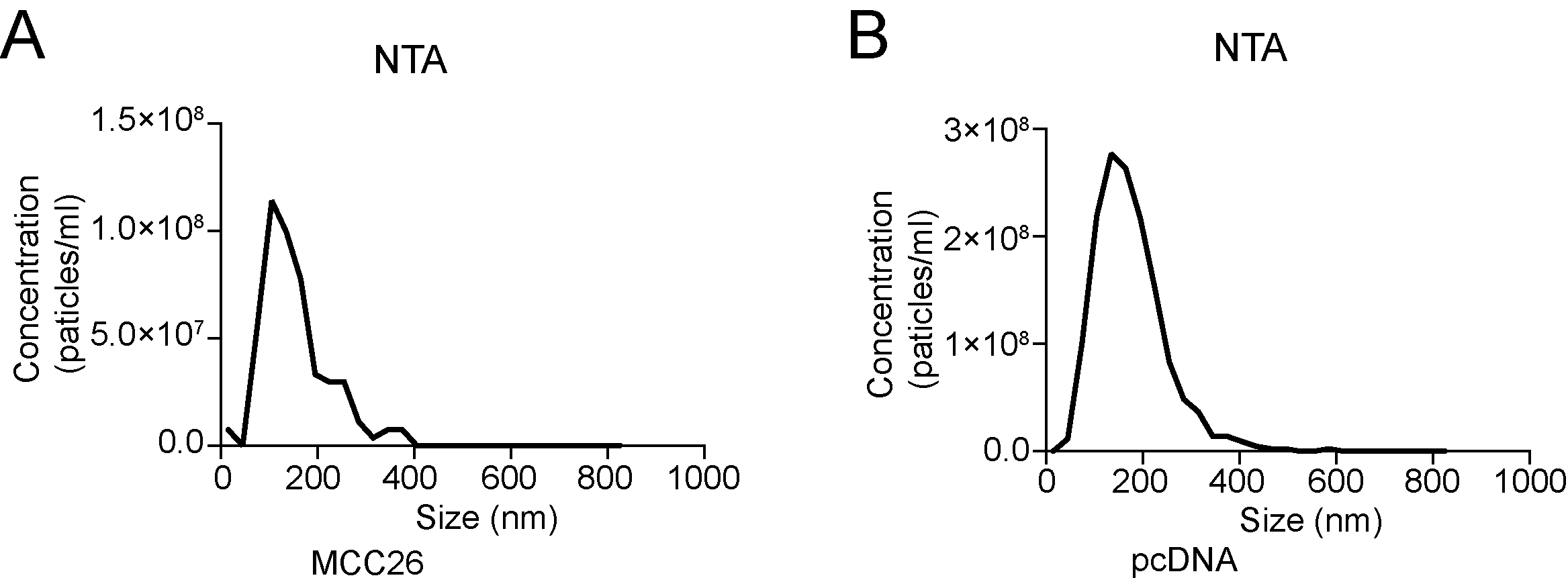

Supplement: S6 Fig — (A-B) Size distribution analysis of total exosomes isolated from MCC26 cells (A) and 293T cells co-transfected with pCDNA vector (B). (TIF) [file ppat.1009582.s006.tif]

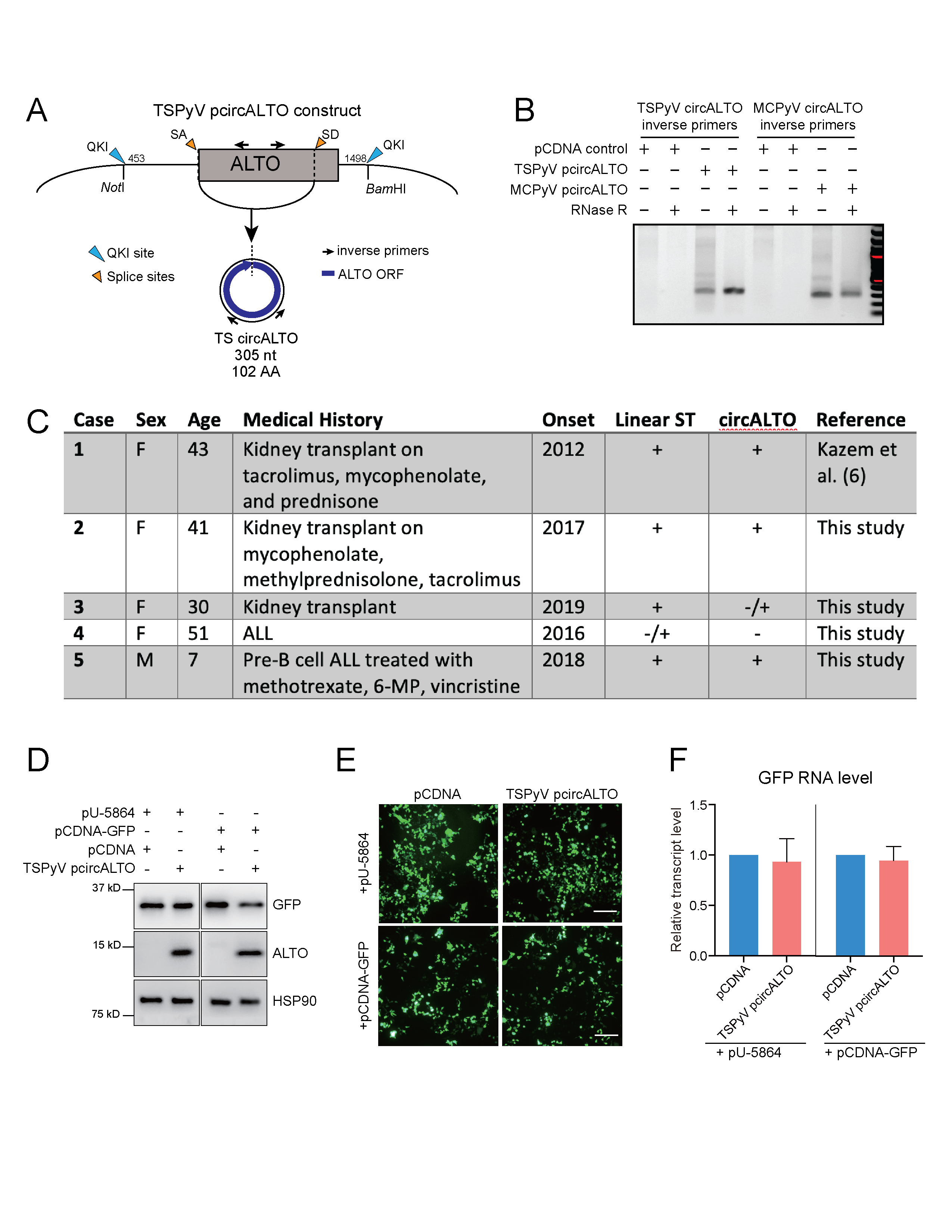

Supplement: S7 Fig — Identification and characterization of TSPyV circALTO. (A) Schematic diagram of TSPyV circALTO expression constructs generated in vitro. The location of QKI sites, ORF, and splice sites were indicated in the diagram. (B) RT-PCR analysis of total RNA from 293T cells co-transfected with MCPyV circALTO or TSPyV circALTO plasmids with and without RNase R treatment. (C) Table summarizing the clinical characteristics and RT-PCR results of TS patients utilized in this study. Only patient 1 has previously been reported. (D) Western blot for GFP and ALTO from 293T cells were co-transfected with the indicated plasmids: either pU-5864 or pCDNA-GFP AND either pCDNA control vector or TSPyV circALTO plasmids. HSP90 serves as the loading control. (E) IF images of 293T cells co-transfected with either pU-5864 or pCDNA-GFP and either pCDNA vector or TSPyV pcircALTO for 48 hours. Scale bar = 200 μm. (F) qRT-PCR of GFP transcripts from 293T cells co-transfected with either pU-5864 or pCDNA-GFP AND either pCDNA or TSPyV circALTO. ACTB served as the internal control. Error bars = SD from a single experiment. Results are representative of 2 independent experiments. (TIF) [file ppat.1009582.s007.tif]

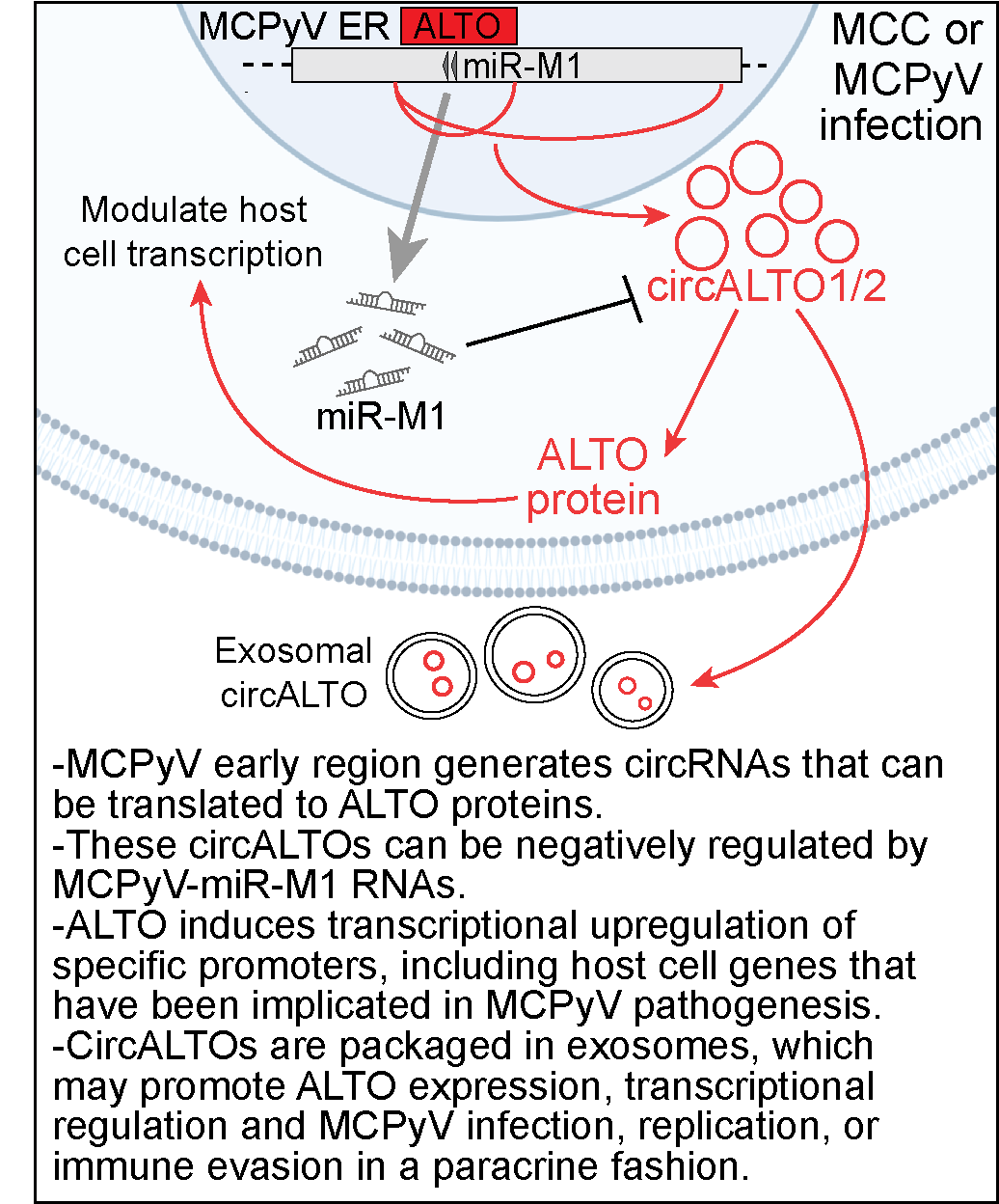

Supplement: S8 Fig — Speculative model for the regulation and function of MCPyV circALTO. (TIF) [file ppat.1009582.s008.tif]
